# Clinical pathway modelling: a literature review

**DOI:** 10.1080/20476965.2019.1652547

**Published:** 2019-09-11

**Authors:** Emma Aspland, Daniel Gartner, Paul Harper

**Affiliations:** School of Mathematics, Cardiff University, Cardiff, UK

**Keywords:** Clinical pathways, literature review, operational research, information systems

## Abstract

Hospital information systems are increasingly used as part of decision support tools for planning at strategic, tactical and operational decision levels. Clinical pathways are an effective and efficient approach in standardising the progression of treatment, to support patient care and facilitate clinical decision making. This literature review proposes a taxonomy of problems related to clinical pathways and explores the intersection between Information Systems (IS), Operational Research (OR) and industrial engineering. A structured search identified 175 papers included in the taxonomy and analysed in this review. The findings suggest that future work should consider industrial engineering integrated with OR techniques, with an aim to improving the handling of multiple scopes within one model, while encouraging interaction between the disjoint care levels and with a more direct focus on patient outcomes. Achieving this would continue to bridge the gap between OR, IS and industrial engineering, for clinical pathways to aid decision support.

## 1. Introduction

Clinical pathways are an effective and efficient way to standardise the progression of treatment, which in effect can reduce the pressure and problems surrounding subject areas that decision makers need to address.

There is a vast scope for what can encompass the term clinical pathway, with numerous ways of formulating, approaching, and modelling them. As far as we are aware, the only literature reviews to explore specifically ‘clinical pathways’ in relation to OR is Elbattah and Molloy [61] and Erdogan and Tarhan [63]. Our paper differs from previous work as we provide a rigorous taxonomy to characterise an abundant work of literature around clinical pathways. Subsequently, we demonstrate the applicability of our taxonomy by classifying the research papers into the different categories.

This paper provides a general overview of the publications surrounding clinical pathways in healthcare, in addition to various detailed classifications of such publications. This enables clarity for any future publications surrounding clinical pathways to identify the current themes and methods used in the literature, and thus identify gaps.

For each figure that displays a key classification result, there is a respective table within Appendix II, which fully details the reference number for each paper within the category.

The remainder of the paper is structured as follows: Section 2 provides a definition of clinical pathways, Section 3 describes the search criteria, Section 4 discusses previous literature reviews. Section 5 then explores a sample of the selected papers to aid understanding of the taxonomy, Section 6 displays the taxonomy results for the literature and Section 7 closes the paper with a discussion and conclusion.

## 2. Definition

The first use of the term ‘clinical pathway’ was in 1985 by Zander, Etheredge, and Bower (1987) at the New England Medical Centre  (De Bleser, Depreitere, de Waele, Vanhaecht, and Vlayen, 2006). Since then, the term has become more frequently used and mutated into multiple terms. For instance, deLuc et al. (2001) found 17 different terms which denoted the concept of ‘clinical pathways’, and discussed that the most common terms were ‘care pathway’, ‘critical pathway’, ‘integrated care pathway’ and ‘care map’.

De Bleser, Depreitere, de Waele, Vanhaecht, and Vlayen (2006) conducted a literature review with the aim to ‘survey the definitions used in describing the concept and to derive key characteristics of clinical pathways’. The authors found 84 different definitions of a clinical pathway between 2000 and 2003.

Kinsman, Rotter, James, Snow, and Willis (2010) conducted a literature review and developed detailed criteria for what should be classified as a clinical pathway and tested this against 260 papers. They developed the following criteria:

- The intervention was a structured multidisciplinary plan of care.
- The intervention was used to channel the translation of guidelines or evidence into local structures.
- The intervention detailed the steps in a course of treatment or care in a plan, pathway, algorithm, guideline, protocol or other ‘inventory of actions’.
- The intervention had time-frames or criteria-based progression (that is, steps were taken if designated criteria were met).
- The intervention aimed to standardise care for a specific clinical problem, procedure or episode of healthcare in a specific population.

If an intervention satisfied the first, and then any three of the remaining four criteria, then it was classified as a clinical pathway.

This is a very detailed definition which clearly describes the features of a clinical pathway.

## 3. Search criteria

A structured search was conducted using the Scopus search engine restricting to years 1998–2018 (November). The keywords were specified to focus on clinical pathway and its main alternative terms as indicated by deLuc et al. (2001): ‘care pathway’, ‘critical pathway’ and ‘care map’. Two further terms were also included, namely ‘anticipated recovery pathways’ and ‘patient pathway’.

The term ‘patient flow’ was not included in our search terms as this review is specifically interested in the structure of well-defined pathways, and patient flow typically relates to the general movement of patients.

We focus the search to journal publications from five categories in the Thomson-Reuters Journal Citation Report (JCR), and as a result only journal articles were returned by the search. The five subject categories are Anaesthesiology (AN), Health Policy and Services (HPS), Industrial Engineering (IE), Medical Informatics (MI) and Operations Research and the Management Sciences (OR/MS), each of which have an impact factor.

These categories were chosen to provide an overview of papers within the Operational Research (OR) area, in addition to highlighting the type of information that is being presented to other areas on this topic. Specifically, these five categories were chosen for the following reasons:

- Anaesthesiology (AN): captures a subgroup of medical journals in which quantitative methods have been published more frequently than in other medical disciplines (e.g. Anaesthesia & Analgesia).
- Health Policy and Services (HPS): captures those journals covering impact on policy decisions and service improvement (e.g. Health Care Management Science and Health Services Research).
- Industrial Engineering (IE): is a quantitative category that covers engineering journals (e.g. Computers and Industrial Engineering or Computers and OR) in which, for example, patient scheduling papers have been published.
- Medical Informatics (MI): to include data mining and healthcare information systems (IS) topics (e.g. *Journal of Medical Systems*).
- Operations Research and the Management Sciences (OR/MS): covers quantitative modelling and journals surrounding OR in healthcare (e.g. *Journal of the Operational Research Society*).

**Figure 1.**
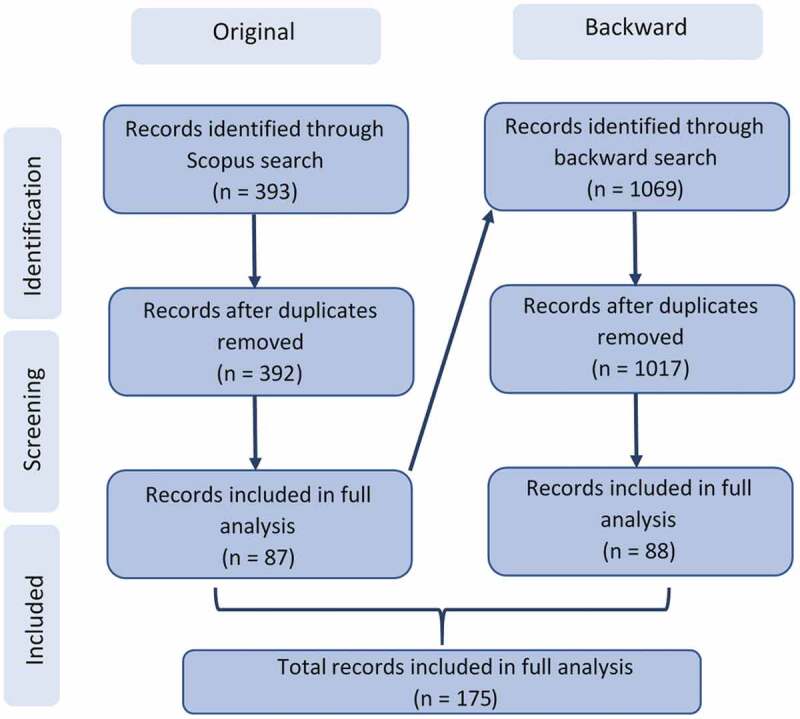
Diagram detailing the search process

Figure 1 shows a diagram detailing the search process.

The screening stage, as displayed in Figure 1, consisted of analysing abstracts of the resulting papers from the search. Any papers that did not refer to a pathway or used only qualitative or statistical methods e.g., interviews or regression respectively, were excluded. The screening stage also excluded papers not available in English. The diagram highlights the use of a backward search, for which the same screening criteria as described above was applied. The final number of records included in the analysis is 175.

## 4. Previous research

Additionally to the 175 papers that were selected, our research revealed 11 papers of notable contribution and 27 literature reviews. All of these papers discuss clinical pathways and the techniques surrounding them, in some form. These are summarised and can be found in Appendix II, Tables A1 and A2, respectively.

Concerning the 11 papers of notable contribution, these discussed guidelines, frameworks, case studies and I.T. artefacts that support clinical pathways.

Of the 27 review papers in Table A2 (Appendix II), six consider process mining or data mining [38, 63, 78, 109, 135, 168], seven consider simulation [2, 105, 132, 173, 175, 176, 208] and three consider stochastic modelling [59, 123, 213].

There are seven papers that use the term ‘clinical pathway’, or its synonyms, in the search terms [61, 63, 132, 156, 173, 175, 189] and two papers use ‘patient flow’ in their search terms [109, 173]. These papers all consider clinical pathways, but most focus on a different primary topic.

There are only two reviews that concern clinical pathways specifically: Elbattah and Molloy [61] and Erdogan and Tarhan [63].

Elbattah and Molloy [61] provide a comprehensive discussion of 22 papers concerning modelling and simulating clinical pathways. Our paper provides a different perspective from [61] as we provide a rigorous taxonomic approach to classifying many papers.

Although Erdogan and Tarhan [63] consider process mining as a primary topic, the amount of consideration and discussion around clinical pathways is vast, indicating 59 papers concerning clinical pathways. The systematic mapping method used is reflective of our method, however as clinical pathways are not the primary consideration, it does not fully consider a discussion of clinical pathways post-discovery. With clinical pathways being the main focus of our paper, it differs from that of Erdogan and Tarhan [63] as we consider a holistic view on clinical pathways.

## 5. Exploration of a sample of papers

This section explores a sample of the papers for the purpose to aid understanding of the classifications in Section 6. These papers have been chosen so as to discuss the widest range of categories, using the smallest sample of papers.

The sample of papers are discussed briefly and their relevant categories are indicated in Table 1

**Table 1.** Summary of categories for sample of papers

No.	Condition	Method	Outcome	Scope	Decision Level
[3]	None	Simulation	Resource & Time	Clinical	Strategic
[14]	None	Optimisation & Heuristics	Cost & Resource	Department	Strategic & Tactical
[19]	Chronic Focus	Simulation	Cost	Clinical	Strategic
[35]	Acute Focus	Simulation	Resource & Time	Clinical	Tactical
[55]	Chronic Applied	Optimisation & Heuristics	Pathway Mapping	Clinical	NA
[97]	Acute Applied	Data Mining or Machine Learning	Pathway Mapping	Clinical	NA
[107]	Surgical Applied	Data Mining or Machine Learning	Pathway Mapping & Patient Progression	Clinical	NA
[116]	Chronic Focus	Simulation	Patient Progression	Hospital	Strategic
[120]	None	Stochastic Modelling	Resource	Disease	Tactical & Operational
[143]	Surgical Focus	Data Mining or Machine Learning	Time	Disease	Operational
[203]	None	Stochastic Modelling & Data Mining or Machine Learning	Legal	Hospital	NA

Ajmi et al., [3] used Business Process Modelling Notation (BPMN) to model the workflows of the patient journey in a Paediatric Emergency Department. The aim was to identify bottlenecks and crowded situation indicators, with noting that delay occurs in the waiting time from the health care request. The study was integrated into the French National Research Agency (ANR) project, titled: ‘Hospital: Optimization, Simulation and avoidance of strain (HOST)’.

Barbagallo et al., [14] used BPMN 2.0 to schedule operating room activity, by room and day through a waiting list database, and applied stochastic modelling to allow optimisation.

Bending et al., [19] used Monte Carlo sampling techniques to estimate the direct cost of bowel cancer services.

Chemweno et al., [35] developed a discrete event simulation on the stay of stroke patients in a stroke unit, specifically diagnosis, to investigate capacity and waiting times.

Du et al., [55] develop a new method of handling clinical pathway variances in Takagi-Sugeno (T-S) fuzzy neural networks (FNNs). Two cases concerning osteosarcoma preoperative chemotherapy are used to validate this method.

Huang et al., [97] used Latent Dirichlet Allocation (LDA) for the purpose of discovering the treatment patterns as a probabilistic combination of clinical activities. The method was then applied as part of experiments to careflow logs concerning intracranial haemorrhage and cerebral infarction.

Konrad et al., [107] developed a method to use message exchanges to automatically establish and compare a patient’s path against a clinical pathway. The method has been applied to a case study in major joint replacement.

Langley et al., [116] developed a discrete event simulation model for the diagnosis of Tuberculosis (TB) to help provide policy makers with the information to decide which tools, and where, they should be implemented for maximum effectiveness.

Lanzarone et al., [120] modelled the home care pathway using a Markov chain, where the future workload of each operator was of interest to support medium and short term resource planning. The model was developed as a simple software application, integrated into the current software used, which supports patient to operator assignment.

Michowski et al., [143] used a Bayesian Belief Network (BBN) to model the radical prostatectomy clinical pathway with an interest in patients length of stay being categorised as ‘met’ or ‘delayed’ given the patient’s outcomes and activities. The research was implemented as an application.

Yang and Hwang [203] utilised clinical pathways, through data-mining using a Markov blanket filter, to facilitate automatic and systematic construction of an adaptable and extensible detection model of fraudulent and system abusive behaviour.

## 6. Classification of literature

This section discusses the taxonomy which classifies the literature, and provides summary statistics.

### 6.1. General characteristics

**Figure 2.**
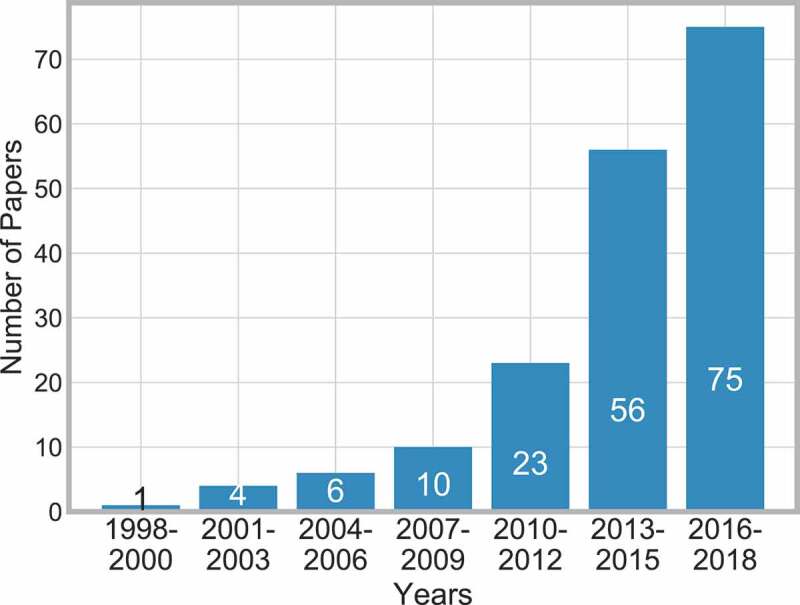
Frequency of publications over time

Figure 2 displays the distribution of the papers across 21 years and shows that the number of publications considering clinical pathways has rapidly increased. This may reflect the growing demand for the use of clinical pathways in practice and thus the need for more in-depth research.

Table 2 shows how the papers were distributed across the world. A paper was classified within a geographical area if it specifically stated that the data or hospital was within that area, or failing that, through any acknowledgements of a hospital in a specific area or the country of the first author was recorded.

**Table 2.** Number of articles by geographical area

Continent	America	Asia	Europe	Other
Total	32	41	90	12

Table 2 shows how Europe has the greatest number of publications relating to clinical pathways, followed by Asia then America. This highlights that research into clinical pathways is of global interest.

This section concludes that research into clinical pathways is growing in popularity across the globe, year on year.

#### 6.1.1. Publication area

**Figure 3.**
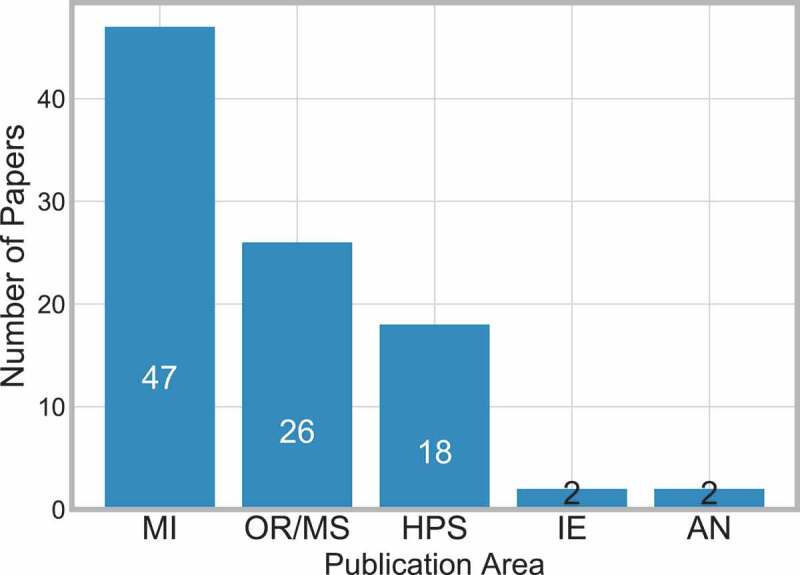
Frequency of publications in JCR category

Figure 3 breaks down the publications by the JCR category which each paper was published under. Again, the five subject categories are Anaesthesiology (AN), Health Policy and Services (HPS), Industrial Engineering (IE), Medical Informatics (MI) and Operations Research and the Management Sciences (OR/MS).

There are 64 papers identified in the backward search, whose ISSN numbers do not relate to any of the five JCR categories, plus a further 16 papers in the backward search that appear to have no ISSN number – and thus also no JCR group. These are not included in Figure 3.

Two journals not included in the JCR categories published multiple papers identified in the search, these are Lecture Notes in Computer Science [20, 48, 85, 87, 98, 112, 179] and Studies in Health Technology and Informatics [5, 74, 76, 94, 121, 136, 210].

It is apparent here that MI is the most popular JCR group followed by OR/MS. Although there were only a few papers in AN and IE, it is beneficial to capture these as they provide another perspective on clinical pathways.

This highlights the need to bridge the gap between MI with OR and IS methods.

#### 6.1.2. Obtaining the pathway

Obtaining the pathway is arguably one of the most important aspects of analysing clinical pathways. As presented in the selected papers, there are two common ways of obtaining this information: either data driven or through collaboration with those who interact with the pathway.

There were many different ways of obtaining data described, including historic [143], billing [85], messages [107] and Electronic Medical Records [88]. Similarly, collaboration took on a number of different forms including, consulting with experts [17], staff [10], patients [136] and through observations [101].

**Figure 4.**
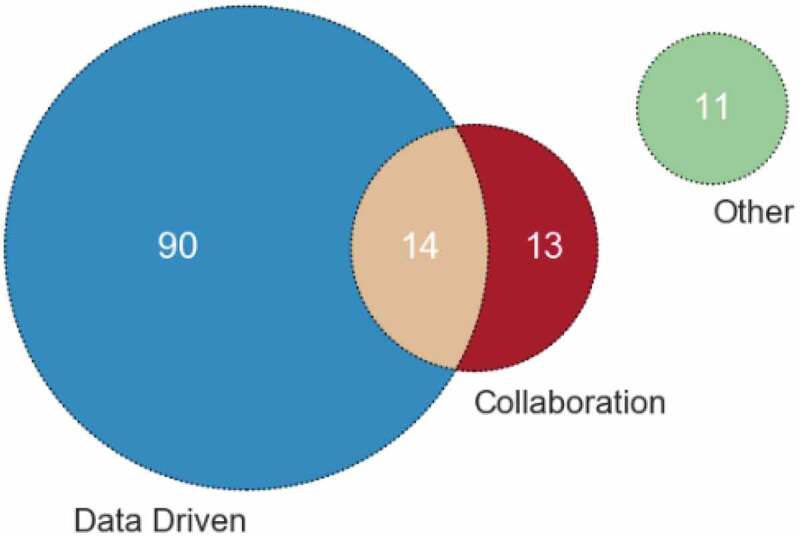
Frequency of papers in each collection method

Figure 4 explores how the information on the pathway was obtained. Forty-seven papers did not clearly specify how they obtained the data and have been classified as unspecified.

Eleven papers specifically stated other methods of collecting the information on the pathway, which are as follows – [19, 22, 41, 82, 116, 169] stated that the information was provided to them in some way, [79] through previous work (also consulted with experts and stakeholders), [128] online user input, and [21, 24, 60] used national guidelines.

The advantage of using data to inform the pathway is that the pathway is derived factually and objectively from actual occurrences of the pathway. The advantage of collaboration with staff and experts is that more information can be gathered about why certain decisions and possible variances from the pathway would occur. Therefore, it is recommended to consider both data driven and collaboration with staff when deriving the pathway, although we observe only 14 papers (8%) in our survey considered both aspects.

It is important to note that only 12 papers [3, 22, 52, 60, 73, 93, 96, 119, 120, 124, 143, 170] state that their research/product was implemented/informed policy – this is only 6.9% of the papers surveyed. Previous reviews have found similar results in regards to implementation (e.g. Brailsford, Harper, Patel, & Pitt, 2009), and therefore this finding highlights the need for more implementation and evaluation. However, caution needs to be considered here as it is possible that some proposed recommendations were/will be eventually implemented but was outside the timeline of the publication.

### 6.2. Medical context

#### 6.2.1. Condition area

The papers selected consider a variety of medical conditions which is either the main focus of the paper, or applied as case study/validation/explanation etc.

There are three condition categories: Acute, Chronic and Surgical, which have been adapted from Zhang, Padman, and Levin (2014). A description of the condition categories are as follows:

- **Acute** – ‘Acute conditions are severe and sudden in onset. This could describe anything from a broken bone to an asthma attack’. (Medline plus, 2018), stroke has been categorised as acute.
- **Chronic** – ‘A chronic condition, is a long-developing syndrome, such as osteoporosis or asthma’. (Medline plus, 2018).
- **Surgical** – Papers where the main condition was a specific surgical procedure are classified here.

The three condition areas have been further categories as either focus or applied:

- **Focus** – The system surrounding the medical condition was the main motivation for the paper.
- **Applied** – The medical condition was considered as a case study or for validation purposes.

**Figure 5.**
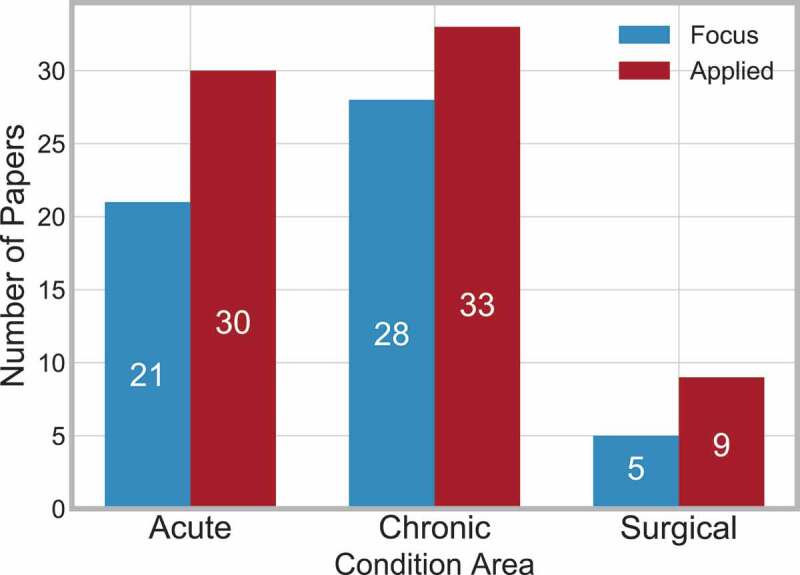
Frequency of papers in each condition area

Figure 5 shows the frequency of papers within each condition area.

Forty-nine papers are not included, as they did not specify a particular condition or considered multiple diagnosis-related groups (DRG).

Chronic conditions are slightly more frequent than acute conditions, and in all three categories, it is more frequent for the condition to be applied rather than the focus of the paper.

#### 6.2.2. Care level

The medical care system is typically split into three sections, Primary, Secondary and Tertiary (NHS providers, 2018), which are as follows:

- **Primary** – First point of contact e.g. General Practitioner or dentist.
- **Secondary** – Can either be elective or emergency care, also known as ‘hospital and community care’.
- **Tertiary** – Highly specialised treatment.

Two other levels can be considered – Home Care and Disease:

- **Home Care** – This is when care is provided to the patient at their own home.
- **Disease** – This concerns understanding how the disease progresses and the care provided progresses alongside.

**Figure 6.**
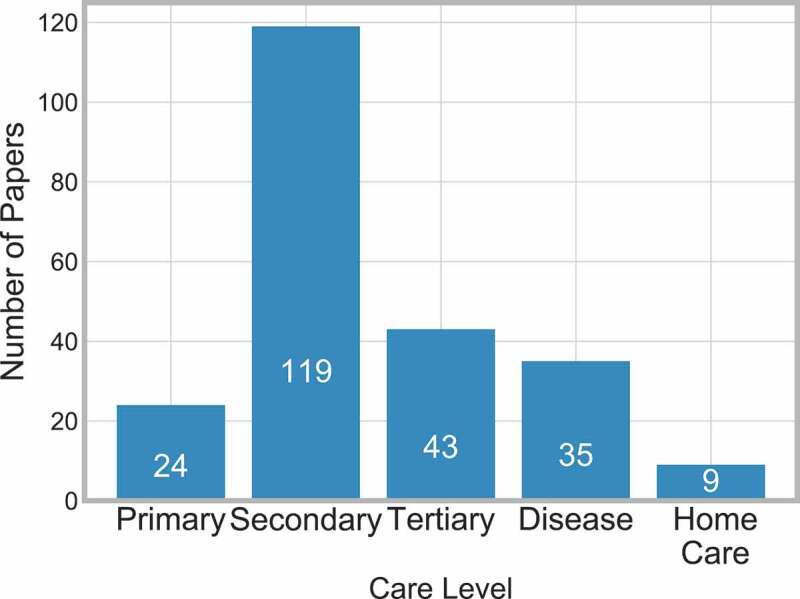
Frequency of papers in each care level

Figure 6 shows the frequency at which each of the five care levels are considered, and displays that secondary care is considered most frequently.

Seven papers [11, 23, 45, 102, 115, 116, 142] consider when the patient is at home and then gets reintroduced into the system in some capacity.

**Figure 7.**
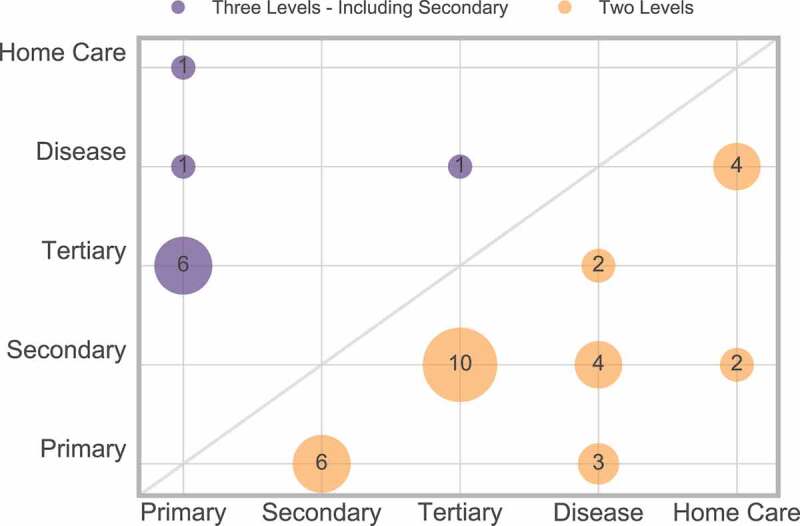
Frequency of multiple care levels

It is important for these systems to work together to allow the patient a smoother journey on the pathway. Within Figure 6, there are 42 papers that consider more than one care level – 31 papers consider two levels, nine consider three levels. The interactions between these levels are displayed in Figure 7.

There are also two papers that consider four levels [16, 145] (primary, secondary, tertiary and home care) which are not displayed in Figure 7.

From Figure 7, we can conclude that only a few papers consider three or more care levels, and therefore research is not providing the full holistic view of the pathway. It is recommended that, when appropriate, future work should make every effort to consider multiple care levels.

**Figure 8.**
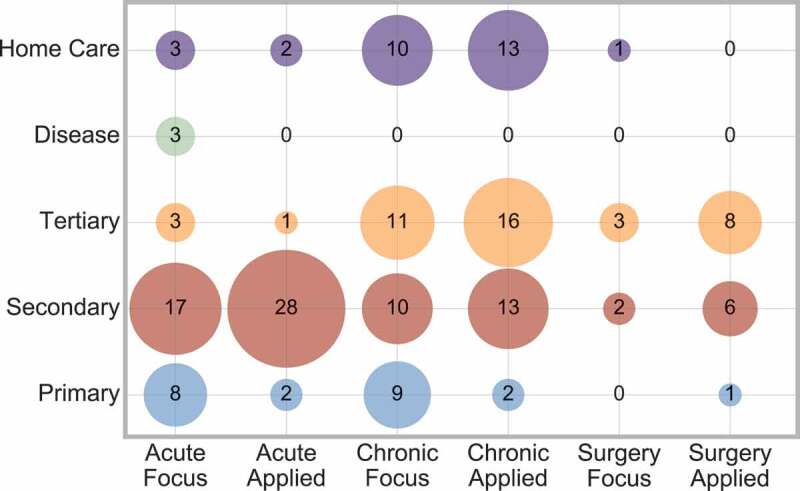
Cross analysis between condition area and care level

The interaction between condition area and care level can be considered, and is displayed in Figure 8.

Figure 8 shows that acute conditions are mainly considered at a secondary care level, whereas chronic conditions are roughly equally divided between secondary, tertiary or home care levels. This implies that chronic conditions have more range to consider different care levels.

#### 6.2.3. Scope

Although a pathway always has a patient in mind, the scope of the focus on the pathway varied greatly from clinical, disease, department and hospital. This scope considers that although it is typical for the activities of concern to revolve around the patients, they may either not be required to be present, or it is the system around the patient that is of interest and not the patient movements themselves.

To explain this further, an example for each type of scope is now discussed.

- Bayer et al., [16] is categorised as ‘Clinical’, as they produce a simulation of the stroke care pathway where, although some activities do not require the patient to be present, the overall focus is on the patient themselves.
- Michalowski et al., [143] is categorised as ‘Disease’, as the activities are related to the patients’ health, e.g. temperature, pain at rest, vital signs etc.
- van de Klundert et al., [187] is categorised as ‘Department’, as they define an activity as ‘an atomic unit of care delivered to the patient, as meaningful to execute or record the care’. They also state that ‘Although we will not explicitly model it, patient need not be present for each of the activities (consider e.g. lab tests)..
- Arnolds and Gartner [8] is categorised as ‘Hospital’, as they focus on improving hospital layout planning by using clinical pathway mining.

**Figure 9.**
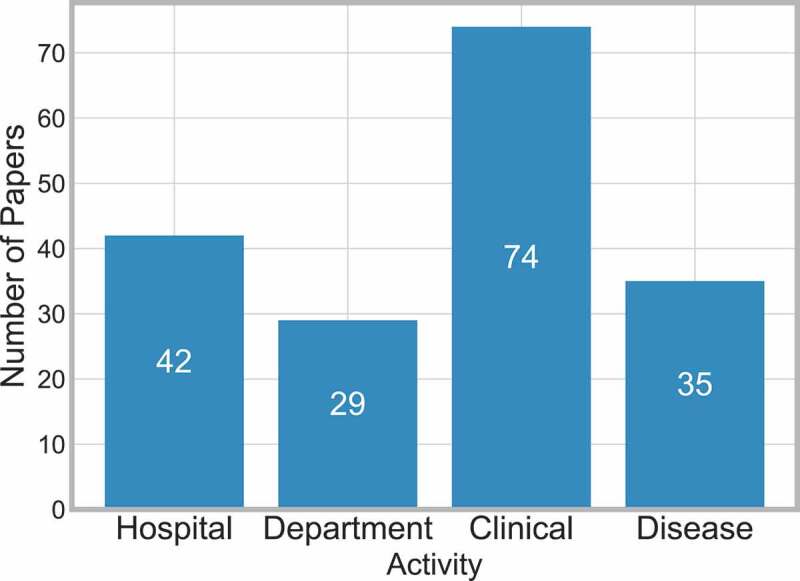
Frequency of papers by scope

Figure 9 displays the frequency of papers in each scope category.

There were only five cases where more than one scope was considered [13, 43, 102, 128, 211]. In all of these cases, both clinical and disease scopes were considered. This was due to the clinical activities being dependent on the progress of disease at particular points e.g. Liu et al., [128] investigate the readmission risk percentage based on the patient activities which differ depending on the diagnosis of the disease.

**Figure 10.**
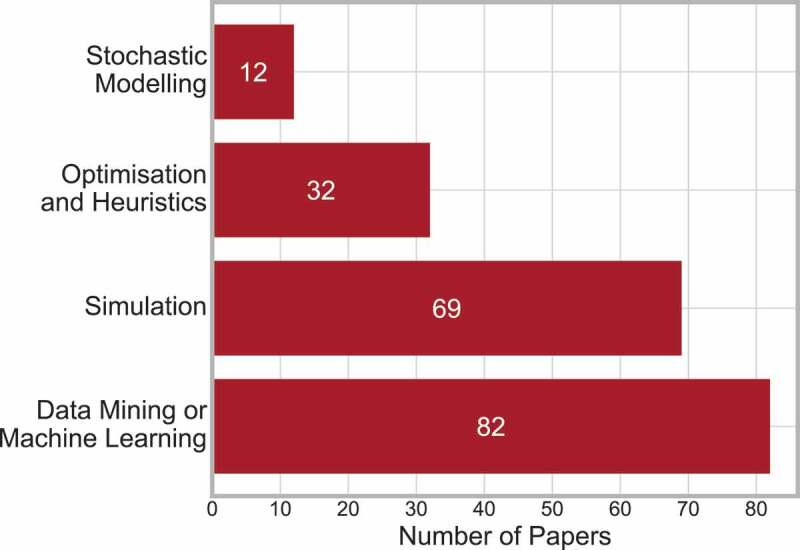
Frequency of papers in each method

From the selected literature, it appears that considering more than one scope area is difficult to carry out in a realistic format, which is not in the form of dummy or pseudo activities. It is believed that this is a limitation of the types of methods (Figure 10 and Table A9) that are considered and thus suggests an opportunity for further work.

### 6.3. Technical context

#### 6.3.1. Method

There are many methods that can be used for clinical pathways, they have been categorised into four groups: Stochastic Modelling, Data Mining or Machine Learning, Simulation, and Optimisation and Heuristics. Further description of what methods are included, but not limited to, in each group are as follows:

- **Stochastic Modelling** – Includes Markov [40] and queueing [190] methods.
- **Data Mining or Machine Learning** – Includes Bayesian techniques and Bayesian Belief Networks [143], machine learning [76] and visualisation [20].
- **Simulation** – Includes discrete-event [79], agent based [129], Monte Carlo [7] and system dynamics [142, 191].
- **Optimisation and Heuristics** – Includes genetic algorithm [57], and mathematical programming, including dynamic [187], mixed-integer [75], mixed-integer linear [31] and goal [167].

Figure 10 displays the frequency of papers in each method group, and indicates that data mining or machine learning was the most popular method to be applied, closely followed by simulation.

**Figure 11.**
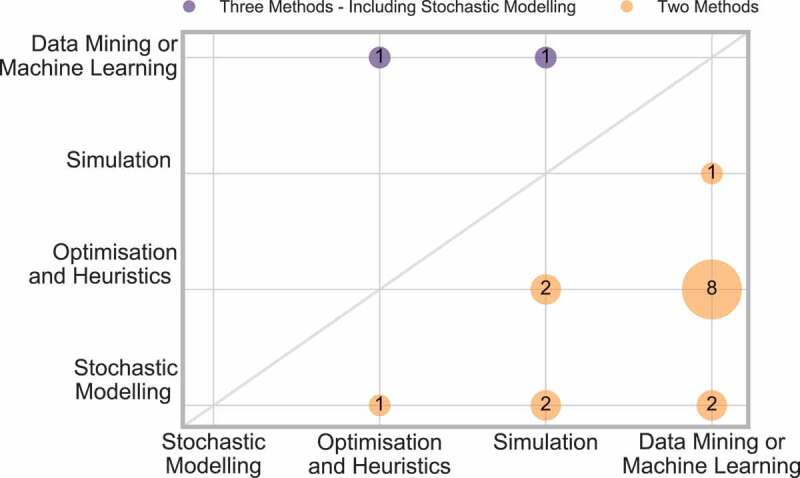
Frequency of papers applying two methods

Eighteen papers were identified as using multiple methods, 16 of those papers applied two methods and two papers applied three methods. This is just 10% of the total selected papers. The combinations of methods applied are displayed in Figure 11.

The majority of these papers use data mining or machine learning along with one other method, and thus shows that those papers using multiple techniques are bridging the gap between OR, IS and industrial engineering.

**Figure 12.**
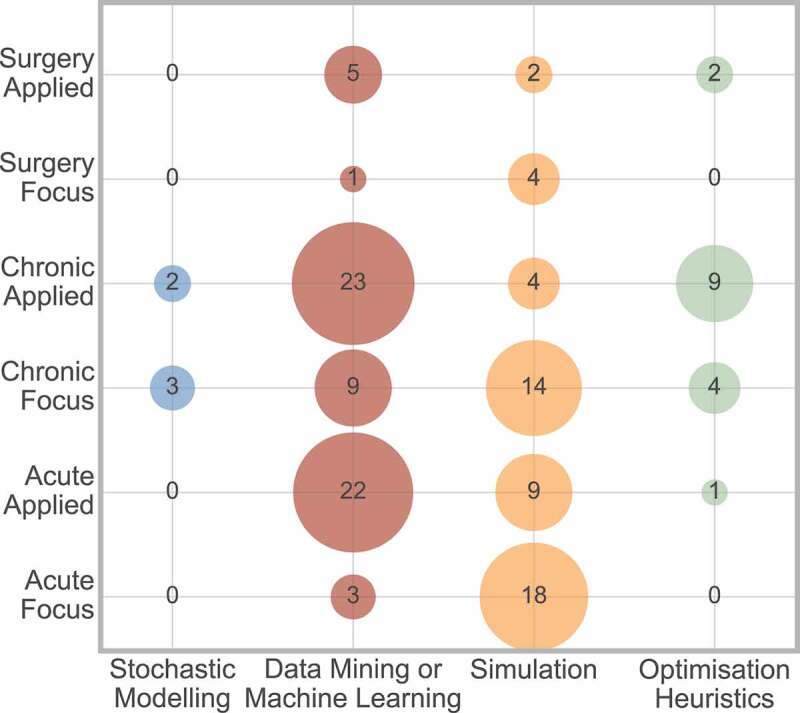
Cross analysis between method and condition area

The interaction between method and condition can be considered, and is displayed in Figure 12.

Figure 12 shows that data mining or machine learning more frequently considers applied conditions, and simulation more frequently has the condition as the focus of the paper, in all three condition areas.

Furthermore, six papers [3, 14, 24, 151, 164, 202] discuss the use of ‘Business Process Modeling Notation’ (BPMN). BPMN is the use of graphical notation for the purpose of illustrating business processes.

Fourteen papers [1, 11, 13, 18, 42, 81, 87, 100, 120, 127, 128, 144, 157, 210] indicate that they develop a type of IT artefact that can be implemented to support the clinical pathways under consideration. These papers are also bridging the gap between OR, IS and industrial engineering.

This highlights that to continue bridging the gap between OR, IS and industrial engineering future work should consider Data Mining and Machine Learning alongside OR techniques, and integrate them whenever possible.

#### 6.3.2. Investigating area

The literature discusses three ways of investigating the pathway: mapped, modelled and improved. A paper is classed as mapping a pathway if it provides some information and process of initially defining the pathway, modelling if it created a model of that pathway, and improved if some scenario analysis, recommendation or support for improvement was made. It is possible for a paper to consider more than one of these investigation areas.

**Figure 13.**
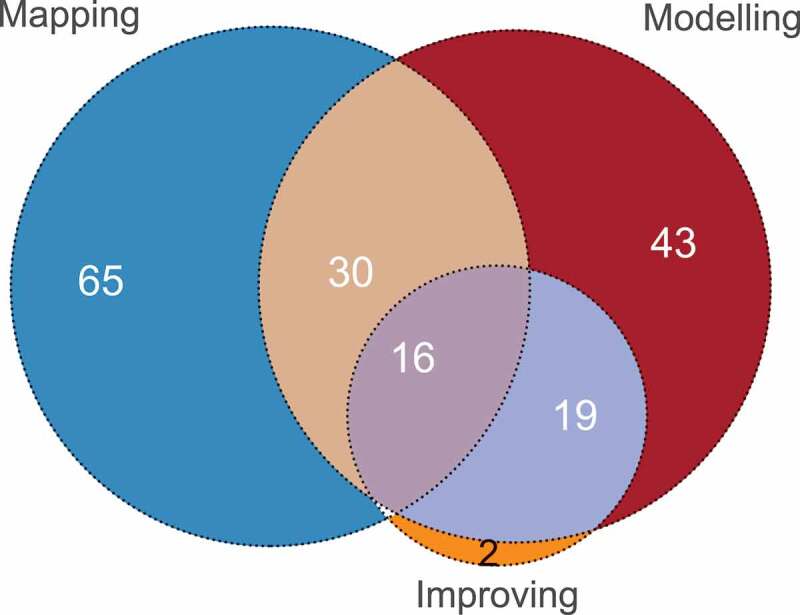
Graph of the interaction between mapping, modelling and improving the pathway

Figure 13 displays the frequency of papers considering each investigation area.

The two papers that were categorised exclusively as improving discussed the development of a web-based tool to aid with clinical pathway usage, and thus did not map or model the pathway. There are no papers that both map and improve the pathway, without also modelling it. This is intuitive, as a model cannot be improved if it was not modelled.

Figure 13 concludes that all three investigation areas are important when considering clinical pathways, and applying all three provides a more complete picture. It is suggested that future work place more focus/importance on improving the pathway and its related outcomes, as this is one of the key advantages of using an OR technique, and can aid decision making.

**Figure 14.**
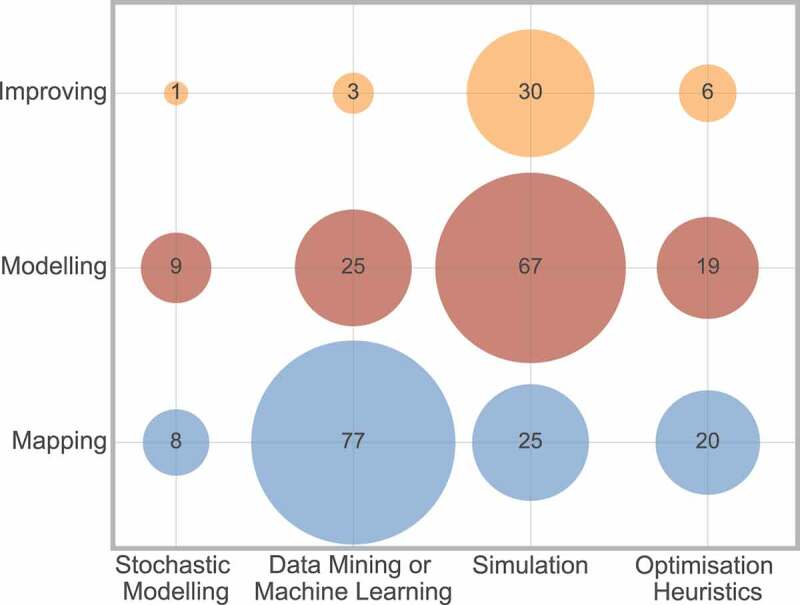
Cross analysis between method and investigation area

The investigation area that is considered is related to the type of method used, as displayed in Figure 14.

Figure 14 displays that the most frequently used techniques to map a pathway are data mining and machine learning, whereas simulation is the most popular technique for considering modelling or improving a pathway.

#### 6.3.3. Outcome

The outcome, main decision variable or indication factor for performance of interest can lead the whole direction of research. The outcomes considered in the literature can be grouped into six categories. A description of the categories is as follows:

- **Legal**: Papers including factors of a legal matter, such as fraud or medical negligence.
- **Patient Progression**: Any factor related to the patient specifically e.g. Quality Adjusted Life Years (QALY), survival, disease progression/management.
- **Cost**: This category includes any paper related to cost.
- **Resource**: Any factor considered to be a resource e.g. MRI Scanner, capacity, staffing levels.
- **Time**: Any factor related to time is included in this category e.g. length of stay, scheduling, waiting times and travel times.
- **Pathway Mapping**: Papers that aimed to establish and map the pathway, including pathway variances are included here.

**Figure 15.**
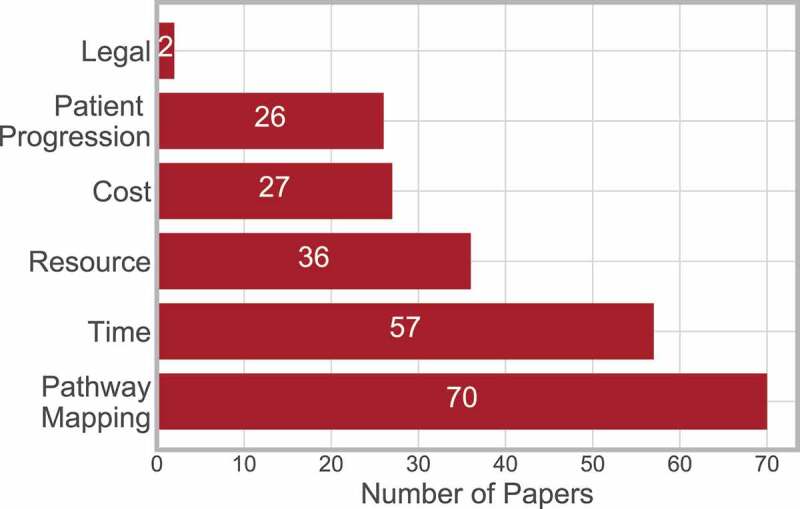
Frequency of papers considering outcome measure

Figure 15 shows the frequency of papers amongst these outcomes. Pathway mapping is the most frequent category, while (excluding legal) patient progression is the least frequent. This may be concerning as the patients are those whose health and lives are effected by all of the outcome factors, and thus should be at the forefront of any outcome considered. Therefore it is recommended that more emphasis should be placed on patient outcomes in a more direct manner.

**Figure 16.**
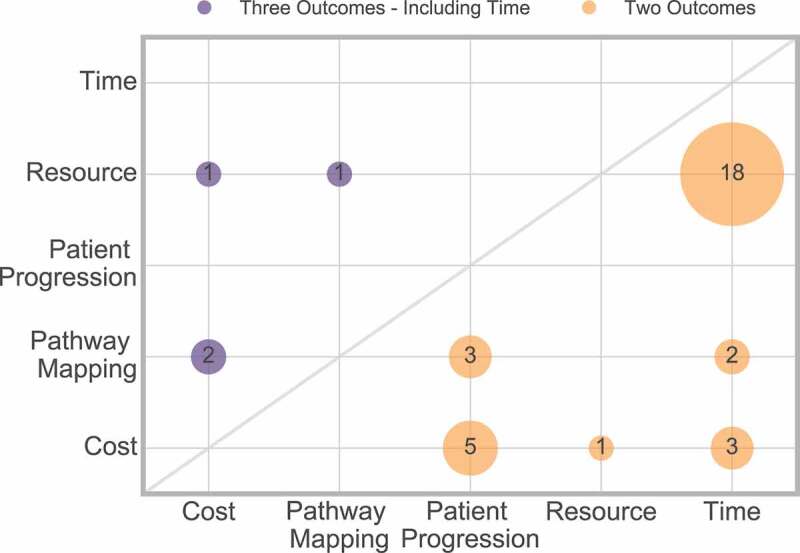
Frequency of interaction of multiple outcomes

Thirty-seven papers considered multiple outcomes, where 32 considered two outcomes, and four considered three outcomes (Figure 16).

Figure 16 shows that time and resource are most frequently considered together, and it is rare to find papers considering more than two outcome measures.

Only one paper considered four outcomes [145] (time, resource cost and patient progression), which is not displayed in Figure 16.

Although an outcome is often regarded as the final result of any research, this also has an impact on the areas surrounding constructing the approach, such as the method or scope considered.

**Figure 17.**
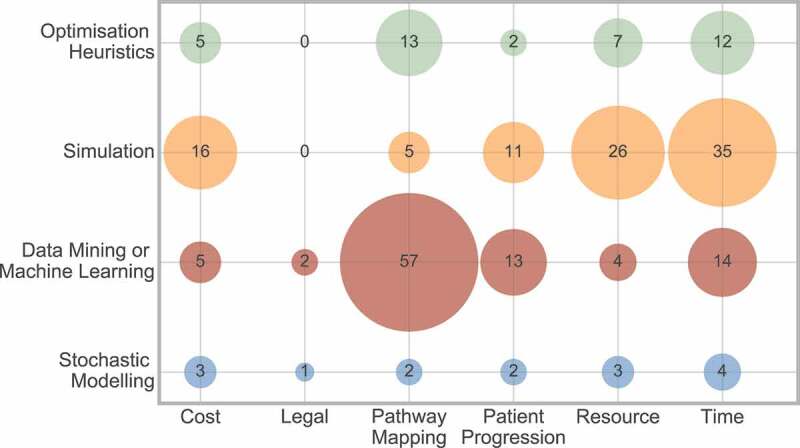
Frequency of interaction between outcome and method

Figure 17 shows the frequencies of the cross analysis between outcome and method. This displays that data mining or machine learning is most frequently used for pathway mapping, whereas simulation is most frequently used to measure cost, resource or time outcome measures.

**Figure 18.**
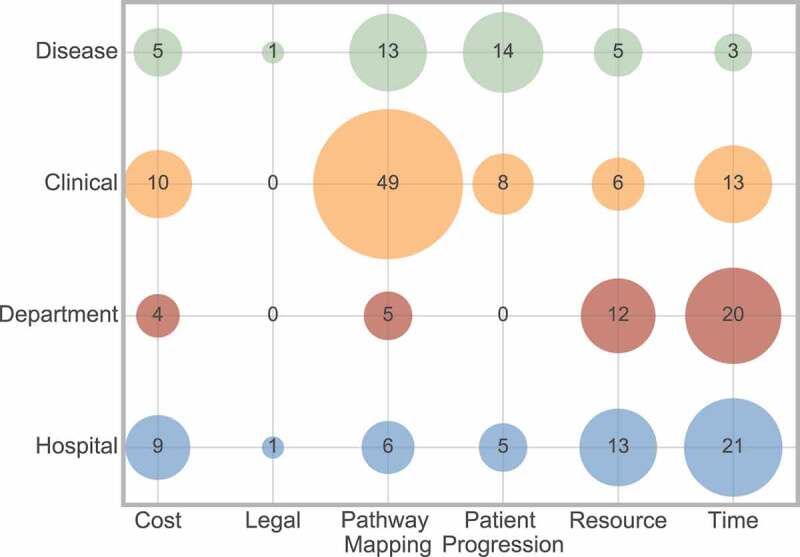
Frequency of interaction between outcome and scope

Figure 18 shows the frequencies of the cross analysis between outcome and scope. It displays that a clinical scope is most frequently used for pathway mapping, whereas resource and time are approximately equally split between hospital and departmental scope.

As an example of the interaction between outcome factor and scope, Barone et al., [15] considered departmental scope in relation to time, resource and cost outcome factors through simulation to plan daily nurse requirements in a stroke unit. In contrast, Uzun Jacobson et al., [186] considered a clinical scope in relation to patient progression outcomes, through discrete-event simulation of hyper-acute stroke care, concerning the percentage of patients receiving thrombolysis.

### 6.4. Planning decisions

Hulshof, Kortbeek, Boucherie, Hans, and Bakker (2012) describes a taxonomic classification of planning decisions in health care in OR/MS. This taxonomy separates the papers into three decision levels: Strategic, Tactical and Operational. A brief description of the decision levels are as follows, however a formal definition of the three decision levels can be found in Hulshof et al. (2012).

- Strategic planning involves structural decision making of the design, dimensioning and development of healthcare. This typically has a long planning horizon e.g. location planning and staffing levels.
- Tactical planning organises the operation of the healthcare delivery system, typically on a mid-term planning horizon, e.g. staff shift scheduling.
- Operational planning executes the routine planning of the healthcare delivery system on a short-term planning horizon e.g. patient-to-appointment scheduling.

**Figure 19.**
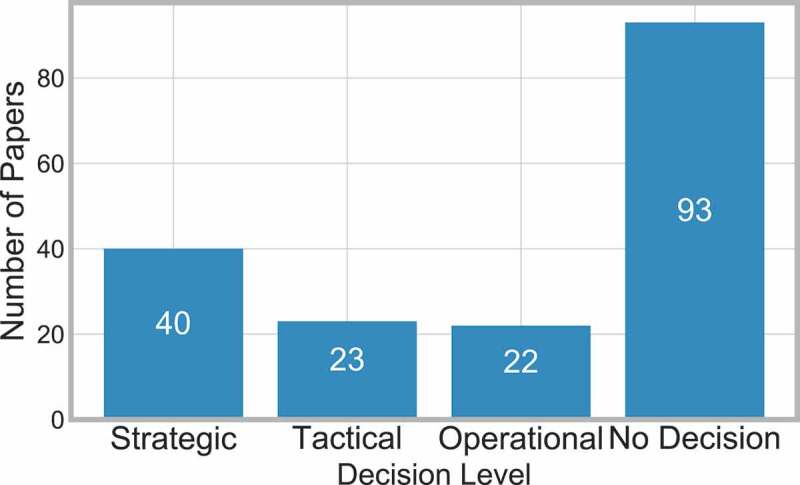
Frequency of papers considering decision level

Figure 19 shows the frequency of the papers in each decision level. This highlights that strategic decisions are considered most frequently out of the three decision levels, however more often than not, there is no decision to consider.

Three papers state that they consider more than one decision level. Barbagallo et al., [14] states that it considers both strategic and tactical decisions, Landa et al., [113] considers tactical and operational decisions and Burdett et al., [31] consider strategic and operational decisions.

This shows that the use of clinical pathways can be used across all the decision levels, from day-to-day decisions to wider policy decisions.

**Figure 20.**
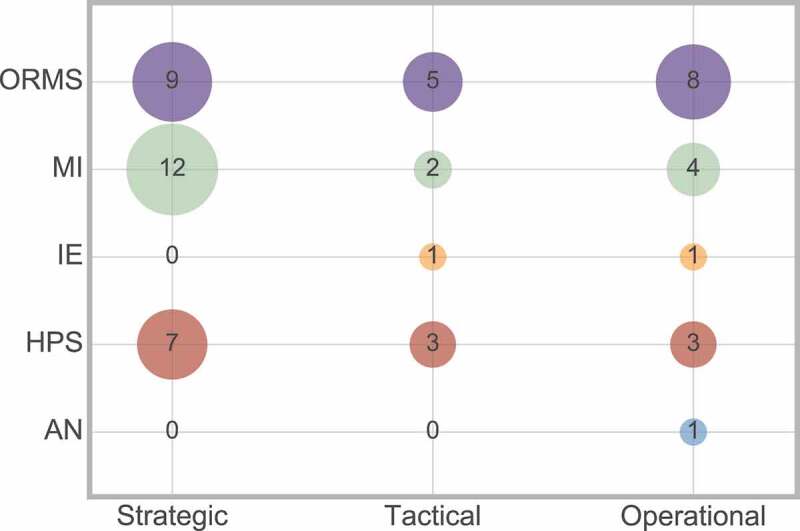
Frequency of interaction between decision level and JCR category

Hulshof et al. (2012) applies the taxonomy for those papers in the OR/MS JCR category, however in this paper we have considered five JCR groups. The cross analysis between decision level and JCR category can be seen in Figure 20.

Figure 20 shows that the decision levels are in fact spread across the five JCR groups, which shows that the Hulshof et al. (2012) decision level taxonomy can be applied to more than just the OR/MS JCR group.

The decision level does impact other aspects of the research that have been previously discussed. Therefore, a cross analysis between the decision level with scope, method, and outcome will now be considered. The cross analyses between decision level and method, and decision level and outcome, both help to explain why such a high number of papers refrain from considering a decision level.

**Figure 21.**
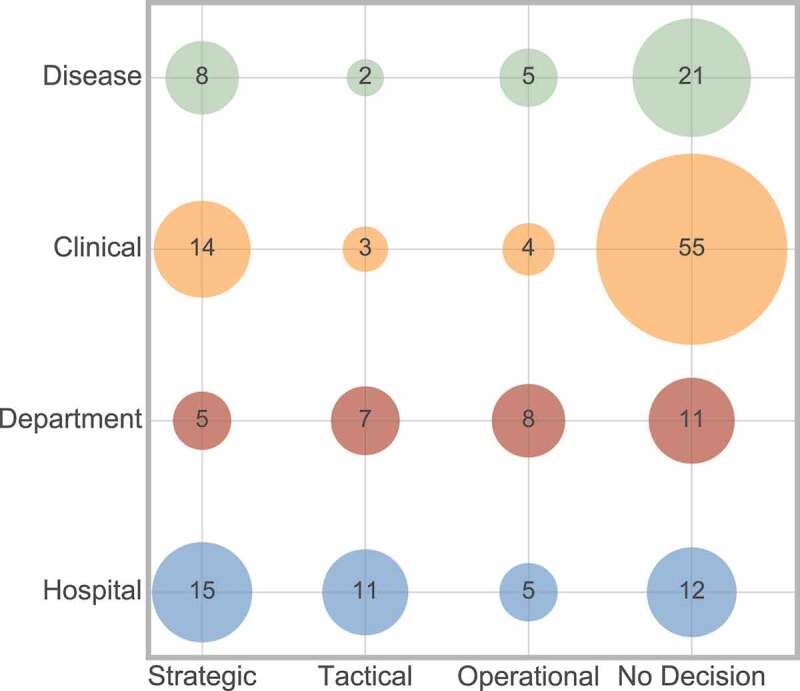
Frequency of interaction between decision level and scope

Firstly, Figure 21 considers the interaction between decision level and scope of the research.

There appears to be an even dispersion of scope across the three decision levels, with strategic being most popular in clinical and disease scope than the other two decision levels. Considering the papers that had no decision level, these are most often concerning clinical scope, but there is also an equal spread between the three remaining scope areas.

**Figure 22.**
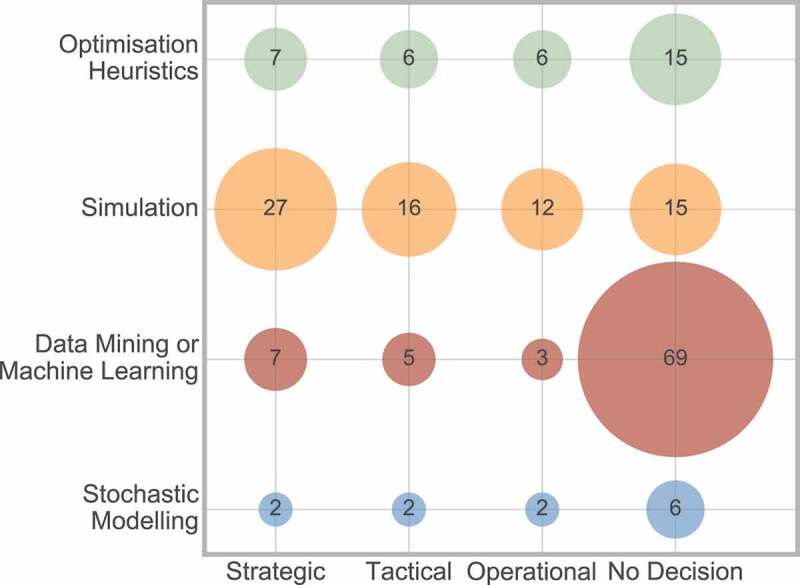
Frequency of interaction between decision level and method

Secondly, Figure 22 considers the interaction between decision level and method.

This shows that simulation is most frequently used across all three decision levels. The interaction between data mining or machine learning and no decision was most commonly observed. This can be explained as this method is most frequently used for mapping a pathway (Figure 14) for reasons such as defining the pathway, and therefore would have no decision associated with this.

**Figure 23.**
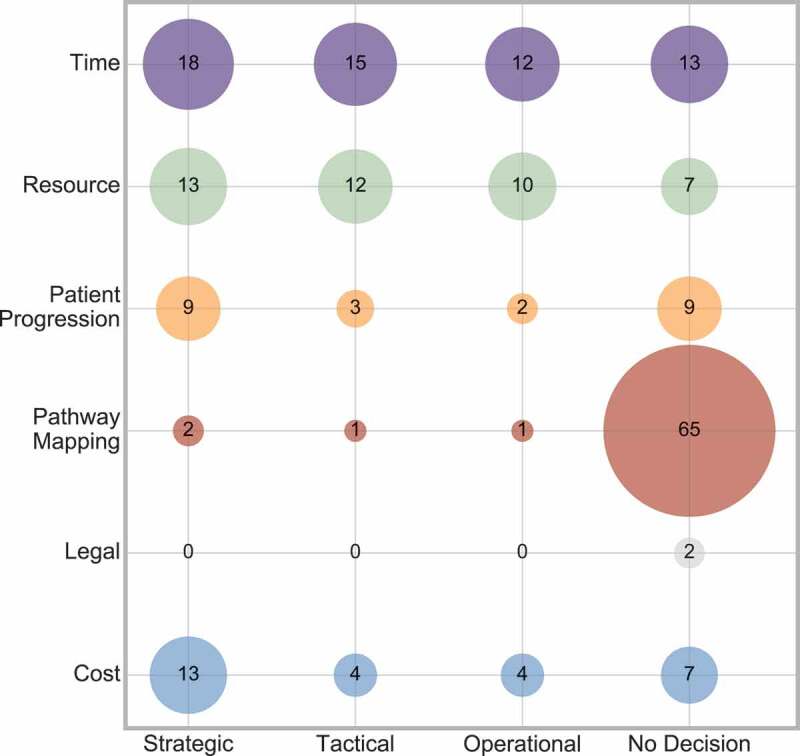
Frequency of interaction between decision level and outcome

The conclusion drawn from the above analyses can be supported when considering the interaction between decision level and outcome measure (Figure 23).

Figure 23 shows that no decision most frequently occurs when the outcome is pathway mapping. Again, all of the outcome measures have an even distribution across all three decision levels, with strategic decisions being slightly more prominent.

## 7. Conclusions

There is a vast scope for what can encompass the term clinical pathway, with numerous ways of formulating, approaching, and modelling these. This paper provides a general overview of the publications surrounding clinical pathways in healthcare. A number of taxonomies have been developed, providing a detailed classification of the publications. This enables clarity for any future publications surrounding clinical pathways to identify the current themes and methods used in the literature, and thus identify gaps.

Section 6 discusses the taxonomy, analysed frequencies, and provided cross analysis where appropriate. Some areas of recommended focus for future work were highlighted in the discussion, and are summarised as follows:

- Careful consideration of publication area, to ensure the information is reaching all communities involved.
- Derive the pathway from both data and collaboration with staff.
- Consider a medical condition, whether in focus or applied application, as this better fits with clinical pathways specifically.
- Include as many care levels as possible (when appropriate) to encourage communication and awareness between them.
- Improve the methods used to allow for multiple types of scope to be considered together.
- Continue to bridge the gap between OR, IS and industrial engineering by considering data mining and machine learning alongside OR techniques, and integrate whenever possible.
- Incorporate all three areas of mapping, modelling and improving the pathway, with particular focus on improving, as this reflects the specialities of OR techniques.
- Greater emphasis on patient outcomes in a more direct manner.
- Specify the decision planning level of focus when appropriate.

Following these recommendations should lead to a more thorough study of the whole clinical pathway. The paper from Monks et al., [145] presents a methodology for simulation modelling of stroke care systems, and captures many of the same recommendations as discussed above, and thus is a notable example of what future research should aim to aspire to.

The inclusion of the cross analysis between the identified taxonomy areas allows those who are considering research to more carefully consider the combinations of these areas, both for quality, appropriateness and discovering areas in which there is a lack of research.

In conclusion, future work should consider industrial engineering and IS integrated with OR techniques, with an aim to improve the handling of multiple scope within one model, while encouraging interaction between the previously disjoint care levels, with a more direct focus on patient outcomes. Achieving this would continue to bridge the gap between OR, IS and industrial engineering, while improving methods for clinical pathways to aid in supporting decisions.

It is important to continue research into clinical pathways, as it is a subject of global interest, whose popularity continues to grow over time.

## Appendix

1. S.R. Abidi and S.S.R. Abidi. An ontological modelling approach to align institution-specific clinical pathways: Towards inter-institution care standardisation. *Proceedings – IEEE Symposium on Computer-Based Medical Systems*, 2012.
2. H.H.A. Afzali, J. Karnon, and J. grey. A critical review of model-based economic studies of depression: Modelling techniques, model structure and data sources. *PharmacoEconomics*, 30(6):461–482, 2012.
3. I. Ajmi, H. Zgaya, L. Gammoudi, S. Hammadi, A. Martinot, R. Beuscart, and J.-M. Renard. Mapping patient path in the paediatric emergency department: A workflow model driven approach. *Journal of Biomedical Informatics*, 54:315–328, 2015.
4. J.M. Albert and S. Nelson. Generalised causal mediation analysis. *Biometrics*, 67(3):1028–1038, 2011.
5. A. Alharbi, A. Bulpitt, and O.A. Johnson. Towards unsupervised detection of process models in healthcare. *Studies in Health Technology and Informatics*, 247:381–385, 2018.
6. M. Andellini, S. Fernandez Riesgo, F. Morolli, M. Ritrovato, P. Cosoli, S. Petruzzellis, and N. Rosso. Experimental application of business process management technology to manage clinical pathways: A pediatric kidney transplantation follow up case. *BMC Medical Informatics and Decision Making*, 17(1), 2017.
7. R. Argiento, A. Guglielmi, E. Lanzarone, and I. Nawajah. A Bayesian framework for describing and predicting the stochastic demand of home care patients. *Flexible Services and Manufacturing Journal*, 28(1–2):254–279, 2016.
8. I.V. Arnolds and D. Gartner. Improving hospital layout planning through clinical pathway mining. *Annals of Operations Research*, 263(1–2):453–477, 2018.
9. Y. Asukai, M. Baldwin, T. Fonseca, A. Gray, L. Mungapen, and D. Price. Improving clinical reality in chronic obstructive pulmonary disease economic modelling: Development and validation of a micro-simulation approach. *PharmacoEconomics*, 31(2):151–161, 2013.
10. N. Bahou, C. Fenwick, G. Anderson, R. van der Meer, and T. Vassalos. Modeling the critical care pathway for cardiothoracic surgery. *Health Care Management Science*, 21(2):192–203, 2018.
11. K. Baker, E. Dunwoodie, R.G. Jones, A. Newsham, O. Johnson, C.P. Price, J. Wolstenholme, J. Leal, P. McGinley, C. Twelves, and G. Hall. Process mining routinely collected electronic health records to define real-life clinical pathways during chemotherapy. *International Journal of Medical Informatics*, 103:32–41, 2017.
12. M. Bakker and K.-L. Tsui. Dynamic resource allocation for efficient patient scheduling: A data-driven approach. *Journal of Systems Science and Systems Engineering*, 26(4):448–462, 2017.
13. P. Balatsoukas, R. Williams, C. Davies, J. Ainsworth, and I. Buchan. User interface requirements for web-based integrated care pathways: Evidence from the evaluation of an online care pathway investigation tool. *Journal of Medical Systems*, 39(11), 2015.
14. S. Barbagallo, L. Corradi, J. De Ville De Goyet, M. Iannucci, I. Porro, N. Rosso, E. Tanfani, and A. Testi. Optimization and planning of operating theatre activities: An original definition of pathways and process modeling. *BMC Medical Informatics and Decision Making*, 15(1), 2015.
15. P. Barone, F. Imbimbo, R. Napoletano, S. Riemma, and D. Sarno. A simulation tool to plan daily nurse requirements. *4th International Workshop on Innovative Simulation for Health Care, IWISH 2015*, pages 61–65, 2015.
16. S. Bayer, C. Petsoulas, B. Cox, A. Honeyman, and J. Barlow. Facilitating stroke care planning through simulation modelling. *Health Informatics Journal*, 16(2):129–143, 2010.
17. S. Ben Othman, H. Zgaya, S. Hammadi, A. Quilliot, A. Martinot, and J.-M. Renard. Agents endowed with uncertainty management behaviors to solve a multiskill healthcare task scheduling. *Journal of Biomedical Informatics*, 64:25–43, 2016.
18. I. Bendavid, Y.N. Marmor, and B. Shnits. Developing an optimal appointment scheduling for systems with rigid standby time under pre-determined quality of service. *Flexible Services and Manufacturing Journal*, 30(1–2):54–77, 2018.
19. M.W. Bending, P. Trueman, K.V. Lowson, H. Pilgrim, P. Tappenden, J. Chilcott, and J. Tappenden. Estimating the direct costs of bowel cancer services provided by the National Health Service in England. *International Journal of Technology Assessment in Health Care*, 26(4):362–369, 2010.
20. J.H. Bettencourt-Silva, G.S. Mannu, and B. de la Iglesia. Visualisation of integrated patient-centric data as pathways: Enhancing electronic medical records in clinical practice. *Lecture Notes in Computer Science (including subseries Lecture Notes in Artificial Intelligence and Lecture Notes in Bioinformatics)*, 9605 LNCS:99–124, 2016.
21. A. Bijlani, A.E. Hebert, M. Davitian, H. May, M. Speers, R. Leung, N.E. Mohamed, H.S. Sacks, and A. Tewari. A multidimensional analysis of prostate surgery costs in the United States: Robotic-assisted versus retropubic radical prostatectomy. *Value in Health*, 19(4):391–403, 2016.
22. C. Born, M. Carbajal, P. Smith, M. Wallace, K. Abbott, S. Adyanthaya, E.A. Boyd, C. Keller, J. Liu, W. New, T. Rieger, B. Winemiller, and R. Woestemeyer. Contract optimization at texas children’s hospital. *Interfaces*, 34(1 SPEC. ISS.):51–58, 2004.
23. J. Bowers, G. Mould, and C. Marshall. Location of services and the impact on healthcare quality: Insights from a simulation of a musculoskeletal physiotherapy service. *Journal of the Operational Research Society*, 66(7):1212–1221, 2015.
24. J. Bowles, M.B. Caminati, and S. Cha. An integrated framework for verifying multiple care pathways. *Proceedings – 11th International Symposium on Theoretical Aspects of Software Engineering, TASE 2017*, 2018-January:1–8, 2018.
25. B.D. Bradley, S.R.C. Howie, T.C.Y. Chan, and Y.-L. Cheng. Estimating oxygen needs for childhood pneumonia in developing country health systems: A new model for expecting the unexpected. *PLoS ONE*, 9(2), 2014.
26. S.C. Brailsford, T.B. Bolt, G. Bucci, T.M. Chaussalet, N.A. Connell, P.R. Harper, J.H. Klein, M. Pitt, and M. Taylor. Overcoming the barriers: A qualitative study of simulation adoption in the NHS. *Journal of the Operational Research Society*, 64(2):157–168, 2013.
27. S.C. Brailsford, V.A. Lattimer, P. Tarnaras, and J.C. Turnbull. Emergency and on-demand health care: Modelling a large complex system. *Journal of the Operational Research Society*, 55(1):34–42, 2004.
28. B. Brown, P. Balatsoukas, R. Williams, M. Sperrin, and I. Buchan. Multi-method laboratory user evaluation of an actionable clinical performance information system: Implications for usability and patient safety. *Journal of Biomedical Informatics*, 77:62–80, 2018.
29. S. Bruzzi, P. Landa, E. Tnfani, and A. Testi. Conceptual modelling of the flow of frail elderly through acute-care hospitals: An evidence-based management approach. *Management Decision*, 56(10):2101–2124, 2018.
30. R.L. Burdett and E. Kozan. An integrated approach for scheduling health care activities in a hospital. *European Journal of Operational Research*, 264(2):756–773, 2018.
31. R.L. Burdett, E. Kozan, M. Sinnott, D. Cook, and Y.-C. Tian. A mixed integer linear programing approach to perform hospital capacity assessments. *Expert Systems with Applications*, 77:170–188, 2017.
32. B. Cardoen and E. Demeulemeester. Capacity of clinical pathways – a strategic multi-level evaluation tool. *Journal of Medical Systems*, 32(6):443–452, 2008.
33. F. Caron, J. Vanthienen, K. Vanhaecht, E. Van Limbergen, J. Deweerdt, and B. Baesens. A process mining-based investigation of adverse events in care processes. *Health Information Management Journal*, 43(1):16–25, 2014.
34. P. Chemweno, L. Brackenier, V. Thijs, L. Pintelon, A. Van Horenbeek, and D. Michiels. Optimising the complete care pathway for cerebrovascular accident patients. *Computers and Industrial Engineering*, 93:236–251, 2016.
35. P. Chemweno, V. Thijs, L. Pintelon, and A. Van Horenbeek. Discrete event simulation case study: Diagnostic path for stroke patients in a stroke unit. *Simulation Modelling Practice and Theory*, 48:45–57, 2014.
36. P. Chemweno, V. Thijs, L. Pintelon, A. Van Horenbeek, and J. Samyn. Simulating the transfer logistics for stroke patients between a stroke unit and the rehabilitation center of a large university hospital. *ILS 2014 – 5th International Conference on Information Systems, Logistics and Supply Chain*, 2014.
37. J. Chen, L. Sun, C. Guo, W. Wei, and Y. Xie. A data-driven framework of typical treatment process extraction and evaluation. *Journal of Biomedical Informatics*, 83:178–195, 2018.
38. J. Chen, W. Wei, C. Guo, L. Tang, and L. Sun. Textual analysis and visualisation of research trends in data mining for electronic health records. *Health Policy and Technology*, 6(4):389–400, 2017.
39. L. Claxton, R. Hodgson, M. Taylor, B. Malcolm, and R. Pulikottil Jacob. Simulation modelling in ophthalmology: Application to cost effectiveness of ranibizumab and aflibercept for the treatment of wet age-related macular degeneration in the United Kingdom. *PharmacoEconomics*, 35(2):237–248, 2017.
40. T. Comans, M. Raymer, S. OLeary, D. Smith, and P. Scuffham. Cost-effectiveness of a physiotherapist-led service for orthopaedic outpatients. *Journal of Health Services Research and Policy*, 19(4):216–223, 2014.
41. T.A. Comans, A.T. Chang, L. Standfield, D. Knowles, S. O’Leary, and M. Raymer. The development and practical application of a simulation model to inform musculoskeletal service delivery in an Australian public health service. *Operations Research for Health Care*, 15:13–18, 2017.
42. D.A. Cook, K.J. Sorensen, J.A. Linderbaum, L.J. Pencille, and D.J. Rhodes. Information needs of generalists and specialists using online best-practice algorithms to answer clinical questions. *Journal of the American Medical Informatics Association*, 24(4):754–761, 2017.
43. K. Cooper, R. Davies, J. Raftery, and P. Roderick. Use of a coronary heart disease simulation model to evaluate the costs and effectiveness of drugs for the prevention of heart disease. *Journal of the Operational Research Society*, 59(9):1173–1181, 2008.
44. J. Coughlan, J. Eatock, and T. Eldabi. Evaluating telemedicine: A focus on patient pathways. *International Journal of Technology Assessment in Health Care*, 22(1):136–142, 2006.
45. G.J. Crane, S.M. Kymes, J.E. Hiller, R. Casson, A. Martin, and J.D. Karnon. Accounting for costs, qalys, and capacity constraints: Using discrete-event simulation to evaluate alternative service delivery and organiZational scenarios for hospital-based glaucoma services. *Medical Decision Making*, 33(8):986–997, 2013.
46. E.A. Crawford, P.J. Parikh, N. Kong, and C.V. Thakar. Analyzing discharge strategies during acute care: A discrete-event simulation study. *Medical Decision Making*, 34(2):231–241, 2014.
47. A. Dagliati, L. Sacchi, A. Zambelli, V. Tibollo, L. Pavesi, J.H. Holmes, and R. Bellazzi. Temporal electronic phenotyping by mining careflows of breast cancer patients. *Journal of Biomedical Informatics*, 66:136–147, 2017.
48. Y. Dauxais, T. Guyet, D. Gross-Amblard, and A. Happe. Discriminant chronicles mining: Application to care pathways analytics. *Lecture Notes in Computer Science (including subseries Lecture Notes in Artificial Intelligence and Lecture Notes in Bioinformatics)*, 10,259 LNAI:234–244, 2017.
49. R. Deja, W. Froelich, G. Deja, and A. Wakulicz-Deja. Hybrid approach to the generation of medical guidelines for insulin therapy for children. *Information Sciences*, 384:157–173, 2017.
50. E. Demir, R. Lebcir, and S. Adeyemi. Modelling length of stay and patient flows: methodological case studies from the uk neonatal care services. *Journal of the Operational Research Society*, 65(4):532–545, 2014.
51. E. Demir and D. Southern. Enabling better management of patients: Discrete event simulation combined with the star approach. *Journal of the Operational Research Society*, 68(5):577–590, 2017.
52. P. Devapriya, C.T.B. Strmblad, M.D. Bailey, S. Frazier, J. Bulger, S.T. Kemberling, and K.E. Wood. Stratbam: A discrete-event simulation model to support strategic hospital bed capacity decisions. *Journal of Medical Systems*, 39(10), 2015.
53. F. Dexter, A. Macario, and E.U. Dexter. Computer simulation of changes in nursing productivity from early tracheal extubation of coronary artery bypass graft patients. *Journal of Clinical Anesthesia*, 10(7):593–598, 1998.
54. G. Du, Z. Jiang, X. Diao, and Y. Yao. Knowledge extraction algorithm for variances handling of cp using integrated hybrid genetic double multi-group cooperative PSO and DPSO. *Journal of Medical Systems*, 36(2):979–994, 2012.
55. G. Du, Z. Jiang, X. Diao, and Y. Yao. Intelligent ensemble t-s fuzzy neural networks with RCDPSO_DM optimization for effective handling of complex clinical pathway variances. *Computers in Biology and Medicine*, 43(6):613–634, 2013.
56. G. Du, Z. Jiang, X. Diao, Y. Ye, and Y. Yao. Variances handling method of clinical pathways based on t-s fuzzy neural networks with novel hybrid learning algorithm. *Journal of Medical Systems*, 36(3):1283–1300, 2012.
57. G. Du, Z. Jiang, Y. Yao, and X. Diao. Clinical pathways scheduling using hybrid genetic algorithm. *Journal of Medical Systems*, 37(3), 2013.
58. R. Dunbar, P. Naidoo, N. Beyers, and I. Langley. Operational modelling: The mechanisms influencing tb diagnostic yield in an XPERTW MTB/RIF-based algorithm. *International Journal of Tuberculosis and Lung Disease*, 21(4):381–388, 2017.
59. S.R. Earnshaw, A.P. Brogan, and C.L. McDade. Model-based cost-effectiveness analyses for prostate cancer chemoprevention: A review and summary of challenges. *PharmacoEconomics*, 31(4):289–304, 2013.
60. J. Eatock, J. Lord, M. Trapero-Bertran, and A. Anagnostou. Discrete event simulation of whole care pathways to estimate cost-effectiveness in clinical guidelines. *Proceedings – Winter Simulation Conference*, 2016-February:1447–1458, 2016.
61. M. Elbattah and O. Molloy. Towards improving modeling and simulation of clinical pathways: Lessons learned and future insights. *SIMULTECH 2015 – 5th International Conference on Simulation and Modeling Methodologies, Technologies and Applications, Proceedings*, pages 508–514, 2015.
62. T.G. Erdogan and A. Tarhan. A goal-driven evaluation method based on process mining for healthcare processes. *Applied Sciences (Switzerland)*, 8(6), 2018.
63. T.G. Erdogan and A. Tarhan. Systematic mapping of process mining studies in healthcare. *IEEE Access*, 6:24,543–25,567, 2018.
64. A.V. Esensoy and M.W. Carter. High-fidelity whole-system patient flow modeling to assess health care transformation policies. *European Journal of Operational Research*, 266(1):221–237, 2018.
65. O. Fennelly, C. Blake, F. Desmeules, D. Stokes, and C. Cunningham. Patient-reported outcome measures in advanced musculoskeletal physiotherapy practice: a systematic review. *Musculoskeletal Care*, 16(1):188–208, 2018.
66. N. Fenton and M. Neil. Comparing risks of alternative medical diagnosis using bayesian arguments. *Journal of Biomedical Informatics*, 43(4):485–495, 2010.
67. R. Feyrer, U. Kunzmann, and M. Weyand. Computer-assisted process simulation: A suitable instrument for process optimization in hospitals [computeruntersttzte prozesssimulation: Ein beitrag zur prozessoptimierung im op]. *Zentralblatt fur Chirurgie*, 131(4):347–353, 2006.
68. R. Feyrer, U. Kunzmann, M. Weyand, and R. Cesnjevar. Process optimization by means of a computerized process simulation model in cardiac surgery. *Disease Management and Health Outcomes*, 14(2):91–97, 2006.
69. A.J. Fong, M. Smith, and A. Langerman. Efficiency improvement in the operating room. *Journal of Surgical Research*, 204(2):371–383, 2016.
70. A.A. Funkner, A.N. Yakovlev, and S.V. Kovalchuk. Data-driven modeling of clinical pathways using electronic health records. *Procedia Computer Science*, 121:835–842, 2017.
71. A.A. Funkner, A.N. Yakovlev, and S.V. Kovalchuk. Towards evolutionary discovery of typical clinical pathways in electronic health records. *Procedia Computer Science*, 119:234–244, 2017.
72. H. Furuhata, K. Araki, T. Ogawa, and M. Ikeda. Effect on completion of clinical pathway for improving clinical indicator: Cases of hospital stay, mortality rate, and comprehensive-volume ratio. *Journal of Medical Systems*, 41(12), 2017.
73. P.H. Garthwaite, J.B. Chilcott, D.J. Jenkinson, and P. Tappenden. Use of expert knowledge in evaluating costs and benefits of alternative service provisions: A case study. *International Journal of Technology Assessment in Health Care*, 24(3):350–357, 2008.
74. D. Gartner, I.V. Arnolds, and S. Nickel. Improving hospital-wide patient scheduling decisions by clinical pathway mining. *Studies in Health Technology and Informatics*, 216:1066, 2015.
75. D. Gartner and R. Kolisch. Scheduling the hospital-wide flow of elective patients. *European Journal of Operational Research*, 233(3):689–699, 2014.
76. D. Gartner and R. Padman. Improving hospital-wide early resource allocation through machine learning. *Studies in Health Technology and Informatics*, 216:315–319, 2015.
77. J.A. George, R. Koka, T.J. Gan, E. Jelin, E.F. Boss, V. Strockbine, D. Hobson, E.C. Wick, and C.L. Wu. Review of the enhanced recovery pathway for children: perioperative anesthetic considerations [les programmes de rcupration rapide pour les enfants: considrations anesthsiques priopratoires]. *Canadian Journal of Anesthesia*, 65(5):569–577, 2018.
78. M. Ghasemi and D. Amyot. Process mining in healthcare: A systematised literature review. *International Journal of Electronic Healthcare*, 9(1):60–88, 2016.
79. J. Gillespie, S. McClean, L. Garg, M. Barton, B. Scotney, and K. Fullerton. A multi-phase des modelling framework for patient-centred care. *Journal of the Operational Research Society*, 67(10):1239–1249, 2016.
80. A.S. Gordon, A.H. Marshall, and M. Zenga. Predicting elderly patient length of stay in hospital and community care using a series of conditional coxian phase-type distributions, further conditioned on a survival tree. *Health Care Management Science*, 21(2):269–280, 2018.
81. S. Guo, K. Xu, R. Zhao, D. Gotz, H. Zha, and N. Cao. Event thread: Visual summarization and stage analysis of event sequence data. *IEEE Transactions on Visualization and Computer Graphics*, 24(1):56–65, 2018.
82. B. Han, L. Jiang, and H. Cai. Abnormal process instances identification method in healthcare environment. *Proc. 10th IEEE Int. Conf. on Trust, Security and Privacy in Computing and Communications, TrustCom 2011, 8th IEEE Int. Conf. on Embedded Software and Systems, ICESS 2011, 6th Int. Conf. on FCST 2011*, pages 1387–1392, 2011.
83. A. Happe and E. Drezen. A visual approach of care pathways from the French nationwide snds database from population to individual records: the epeps toolbox. *Fundamental and Clinical Pharmacology*, 32(1):81–84, 2018.
84. L. He, S. Chalil Madathil, A. Oberoi, G. Servis, and M.T. Khasawneh. A systematic review of research design and modeling techniques in inpatient bed management. *Computers and Industrial Engineering*, 2018.
85. K. Helbig, M. Rmer, and T. Mellouli. A clinical pathway mining approach to enable scheduling of hospital relocations and treatment services. *Lecture Notes in Computer Science (including subseries Lecture Notes in Artificial Intelligence and Lecture Notes in Bioinformatics)*, 9253:242–250, 2015.
86. Z.M. Hira and D.F. Gillies. Identifying significant features in cancer methylation data using gene pathway segmentation. *Cancer Informatics*, 15:189–198, 2016.
87. H. Huang, T. Jin, and J. Wang. Extracting clinical-event-packages from billing data for clinical pathway mining. *Lecture Notes in Computer Science (including subseries Lecture Notes in Artificial Intelligence and Lecture Notes in Bioinformatics)*, 10,219 LNCS:19–31, 2017.
88. Z. Huang, W. Dong, P. Bath, L. Ji, and H. Duan. On mining latent treatment patterns from electronic medical records. *Data Mining and Knowledge Discovery*, 29(4):914–949, 2015.
89. Z. Huang, W. Dong, H. Duan, and H. Li. Similarity measure between patient traces for clinical pathway analysis: Problem, method, and applications. *IEEE Journal of Biomedical and Health Informatics*, 18(1):4–14, 2014.
90. Z. Huang, W. Dong, L. Ji, and H. Duan. Predictive monitoring of clinical pathways. *Expert Systems with Applications*, 56:227–241, 2016.
91. Z. Huang, W. Dong, L. Ji, C. Gan, X. Lu, and H. Duan. Discovery of clinical pathway patterns from event logs using probabilistic topic models. *Journal of Biomedical Informatics*, 47:39–57, 2014.
92. Z. Huang, W. Dong, L. Ji, C. He, and H. Duan. Incorporating comorbidities into latent treatment pattern mining for clinical pathways. *Journal of Biomedical Informatics*, 59:227–239, 2016.
93. Z. Huang, W. Dong, L. Ji, L. Yin, and H. Duan. On local anomaly detection and analysis for clinical pathways. *Artificial Intelligence in Medicine*, 65(3):167–177, 2015.
94. Z. Huang, C. Gan, X. Lu, and H. Huan. Mining the changes of medical behaviors for clinical pathways. *Studies in Health Technology and Informatics*, 192(1–2):117–121, 2013.
95. Z. Huang, Z. Ge, W. Dong, K. He, and H. Duan. Probabilistic modeling personalized treatment pathways using electronic health records. *Journal of Biomedical Informatics*, 86:33–48, 2018.
96. Z. Huang, X. Lu, and H. Duan. On mining clinical pathway patterns from medical behaviors. *Artificial Intelligence in Medicine*, 56(1):35–50, 2012.
97. Z. Huang, X. Lu, and H. Duan. Latent treatment pattern discovery for clinical processes. *Journal of Medical Systems*, 37(2), 2013.
98. Z. Huang, X. Lu, and H. Duan. Similarity measuring between patient traces for clinical pathway analysis. *Lecture Notes in Computer Science (including subseries Lecture Notes in Artificial Intelligence and Lecture Notes in Bioinformatics)*, 7885 LNAI:268–272, 2013.
99. Z. Huang, X. Lu, H. Duan, and W. Fan. summarizing clinical pathways from event logs. *Journal of Biomedical Informatics*, 46(1):111–127, 2013.
100. J.E. Hurwitz, J.A. Lee, K.K. Lopiano, S.A. McKinley, J. Keesling, and J.A. Tyndall. A flexible simulation platform to quantify and manage emergency department crowding. *BMC Medical Informatics and Decision Making*, 14(1), 2014.
101. P. Joranger, A. Nesbakken, G. Hoff, H. Sorbye, A. Oshaug, and E. Aas. Modeling and validating the cost and clinical pathway of colorectal cancer. *Medical Decision Making*, 35(2):255–265, 2015.
102. J. Karnon. Alternative decision modelling techniques for the evaluation of health care technologies: Markov processes versus discrete event simulation. *Health Economics*, 12(10):837–848, 2003.
103. J. Karnon and T. Jones. A stochastic economic evaluation of letrozole versus tamoxifen as a first-line hormonal therapy: For advanced breast cancer in postmenopausal patients. *PharmacoEconomics*, 21(7):513–525, 2003.
104. T.M. Kashner, T.J. Carmody, T. Suppes, A.J. Rush, M.L. Crismon, A.L. Miller, M. Toprac, and M. Trivedi. Catching up on health outcomes: The texas medication algorithm project. *Health Services Research*, 38(1 I):311–331, 2003.
105. M. Keshtkaran, J. Hearne, B. Abbasi, and L. Churilov. Stroke care systems: Can simulation modeling catch up with the recent advances in stroke treatment? *Proceedings – Winter Simulation Conference*, 2016-February:1379–1390, 2016.
106. L.M. Kolarczyk, H. Arora, M.W. Manning, D.A. Zvara, and R.S. Isaak. Defining value-based care in cardiac and vascular anesthesiology: The past, present, and future of perioperative cardiovascular care. *Journal of Cardiothoracic and Vascular Anesthesia*, 32(1):512–521, 2018.
107. R. Konrad, B. Tulu, and M. Lawley. Monitoring adherence to evidence-based practices: A method to utilize hl7 messages from hospital information systems. *Applied Clinical Informatics*, 4(1):126–143, 2013.
108. S.V. Kovalchuk, A.A. Funkner, O.G. Metsker, and A.N. Yakovlev. Simulation of patient flow in multiple healthcare units using process and data mining techniques for model identification. *Journal of Biomedical Informatics*, 82:128–142, 2018.
109. A.P. Kurniati, O. Johnson, D. Hogg, and G. Hall. Process mining in oncology: A literature review. *Proceedings of the 6th International Conference on Information Communication and Management, ICICM 2016*, pages 291–297, 2016.
110. M.M.H. Lahr, D.-J. Van Der Zee, G.-J. Luijckx, P.C.A.J. Vroomen, and E. Buskens. A simulation-based approach for improving utilization of thrombolysis in acute brain infarction. *Medical Care*, 51(12):1101–1105, 2013.
111. M.M.H. Lahr, D.-J. Van Der Zee, G.-J. Luijckx, P.C.A.J. Vroomen, and E. Buskens. Centralising and optimising decentralised stroke care systems: a simulation study on short-term costs and effects. *BMC Medical Research Methodology*, 17(1):1–12, 2017.
112. G.T. Lakshmanan, S. Rozsnyai, and F. Wang. Investigating clinical care pathways correlated with outcomes. *Lecture Notes in Computer Science (including subseries Lecture Notes in Artificial Intelligence and Lecture Notes in Bioinformatics)*, 8094 LNCS:323–338, 2013.
113. P. Landa, M. Sonnessa, E. Tnfani, and A. Testi. Multiobjective bed management considering emergency and elective patient flows. *International Transactions in Operational Research*, 25(1):91–110, 2018.
114. D.C. Lane and E. Husemann. System dynamics mapping of acute patient flows. *Journal of the Operational Research Society*, 59(2):213–224, 2008.
115. I. Langley, E. Adams, B. Doulla, and S.B. Squire. Operational modelling to guide implementation and scale-up of diagnostic tests within the health system: Exploring opportunities for parasitic disease diagnostics based on example application for tuberculosis. *Parasitology*, 141(14):1795–1802, 2014.
116. I. Langley, B. Doulla, H.-H. Lin, K. Millington, and B. Squire. Modelling the impacts of new diagnostic tools for tuberculosis in developing countries to enhance policy decisions. *Health Care Management Science*, 15(3):239–253, 2012.
117. I. Langley, H.-H. Lin, S. Egwaga, B. Doulla, C.-C. Ku, M. Murray, T. Cohen, and S.B. Squire. Assessment of the patient, health system, and population effects of Xpert MTP/RIF and alternative diagnostics for tuberculosis in Tanzania: An integrated modelling approach. *The Lancet Global Health*, 2(10):e581–e591, 2014.
118. E. Lanzarone and A. Matta. The nurse-to-patient assignment problem in home care services. *International Series in Operations Research and Management Science*, 173:121–139, 2012.
119. E. Lanzarone, A. Matta, and E. Sahin. Operations management applied to home care services: The problem of assigning human resources to patients. *IEEE Transactions on Systems, Man, and Cybernetics Part A:Systems and Humans*, 42(6):1346–1363, 2012.
120. E. Lanzarone, A. Matta, and G. Scaccabarozzi. A patient stochastic model to support human resource planning in home care. *Production Planning and Control*, 21(1):3–25, 2010.
121. X. Li, H. Liu, J. Mei, Y. Yu, and G. Xie. Mining temporal and data constraints associated with outcomes for care pathways. *Studies in Health Technology and Informatics*, 216:711–715, 2015.
122. Y. Li, J.A. Schneider, and D.A. Bennett. Estimation of the mediation effect with a binary mediator. *Statistics in Medicine*, 26(18):3398–3414, 2007.
123. M.E. Lim, T. Nye, J.M. Bowen, J. Hurley, R. Goeree, and J.-E. Tarride. Mathematical modeling: The case of emergency department waiting times. *International Journal of Technology Assessment in Health Care*, 28(2):93–109, 2012.
124. F.-R. Lin, S.-C. Chou, S.-M. Pan, and Y.-M. Chen. Mining time dependency patterns in clinical pathways. *International Journal of Medical Informatics*, 62(1):11–25, 2001.
125. Y.C. Lin. Development of an ontology-based flexible clinical pathway system. *WSEAS Transactions on Information Science and Applications*, 6(12):1941–1952, 2009.
126. J. Lismont, A.-S. Janssens, I. Odnoletkova, S. vanden Broucke, F. Caron, and J. Vanthienen. A guide for the application of analytics on healthcare processes: A dynamic view on patient pathways. *Computers in Biology and Medicine*, 77:125–134, 2016.
127. J. Liu, Z. Huang, X. Lu, and H. Duan. An ontology-based real-time monitoring approach to clinical pathway. *Proceedings – 2014 7th International Conference on BioMedical Engineering and Informatics, BMEI 2014*, pages 756–761, 2014.
128. R. Liu, R.V. Srinivasan, K. Zolfaghar, S.-C. Chin, S.B. Roy, A. Hasan, and D. Hazel. Pathway-finder: An interactive recommender system for supporting personalized care pathways. *IEEE International Conference on Data Mining Workshops, ICDMW*, 2015-January:1219–1222, 2015.
129. Z. Liu, D. Rexachs, F. Epelde, and E. Luque. An agent-based model for quantitatively analyzing and predicting the complex behavior of emergency departments. *Journal of Computational Science*, 21:11–23, 2017.
130. J. Lord, S. Willis, J. Eatock, P. Tappenden, M. Trapero-Bertran, A. Miners, C. Crossan, M. Westby, A. Anagnostou, S. Taylor, I. Mavranezouli, D. Wonderling, P. Alderson, and F. Ruiz. Economic modelling of diagnostic and treatment pathways in national institute for health and care excellence clinical guidelines: The modelling algorithm pathways in guidelines (mapguide) project. *Health Technology Assessment*, 17(58):1–150, 2013.
131. R.M. Luque-Baena, D. Urda, M. Gonzalo Claros, L. Franco, and J.M. Jerez. Robust gene signatures from microarray data using genetic algorithms enriched with biological pathway keywords. *Journal of Biomedical Informatics*, 49:32–44, 2014.
132. M. Mahdavi, T. Malmstrm, J. van de Klundert, S. Elkhuizen, and J. Vissers. Generic operational models in health service operations management: A systematic review. *Socio-Economic Planning Sciences*, 47(4):271–280, 2013.
133. K. Maheshwari, J. Cywinski, P. Mathur, III Cummings, K.C., R. Avitsian, T. Crone, D. Liska, F.X. Campion, K. Ruetzler, and A. Kurz. Identify and monitor clinical variation using machine intelligence: a pilot in colorectal surgery. *Journal of Clinical Monitoring and Computing*, 2018.
134. M. Maliapen and B.C. Dangerfield. A system dynamics-based simulation study for managing clinical governance and pathways in a hospital. *Journal of the Operational Research Society*, 61(2):255–264, 2010.
135. M.M. Malik, S. Abdallah, and M. Alaraj. Data mining and predictive analytics applications for the delivery of healthcare services: a systematic literature review. *Annals of Operations Research*, 270(1–2):287–312, 2018.
136. R. Mans, H. Schonenberg, G. Leonardi, S. Panzarasa, A. Cavallini, S. Quaglini, and W. Van Der Aalst. Process mining techniques: An application to stroke care. *Studies in Health Technology and Informatics*, 136:573–578, 2008.
137. J. Marynissen and E. Demeulemeester. Literature review on multi-appointment scheduling problems in hospitals. *European Journal of Operational Research*, 272(2):407–419, 2019.
138. S. McClean, L. Garg, B. Meenan, and P. Millard. Using markov models to find interesting patient pathways. *Proceedings – IEEE Symposium on Computer-Based Medical Systems*, pages 713–718, 2007.
139. S. McClean, T. Young, D. Bustard, P. Millard, and M. Barton. Discovery of value streams for lean healthcare. *2008 4th International IEEE Conference Intelligent Systems, IS 2008*, 1:32–38, 2008.
140. J. Meier, A. Dietz, A. Boehm, and T. Neumuth. Predicting treatment process steps from events. *Journal of Biomedical Informatics*, 53:308–319, 2015.
141. A. Meisami, J. Deglise-Hawkinson, M.E. Cowen, and M.P. van Oyen. Data-driven optimization methodology for admission control in critical care units. *Health Care Management Science*, pages 1–18, 2018.
142. R. Meskarian, M.L. Penn, T. Monks, M.A. Taylor, J. Klein, S.C. Brailsford, and P.R. Benson. Utilisation of health and social care services by the over 65s population. a system dynamics study. *Proceedings of the Operational Research Society Simulation Workshop 2016, SW 2016*, pages 218–227, 2016.
143. W. Michalowski, S. Wilk, A. Thijssen, and M. Li. Using a bayesian belief network model to categorize length of stay for radical prostatectomy patients: Using a Bayesian belief network to categorize los. *Health Care Management Science*, 9(4):341–348, 2006.
144. O. Mohammed and R. Benlamri. Developing a semantic web model for medical differential diagnosis recommendation. *Journal of Medical Systems*, 38(10), 2014.
145. T. Monks, K. Pearn, and M. Allen. Simulation of stroke care systems. *Proceedings – Winter Simulation Conference*, 2016-February:1391–1402, 2016.
146. T. Monks, M. Pearson, M. Pitt, K. Stein, and M.A. James. Evaluating the impact of a simulation study in emergency stroke care. *Operations Research for Health Care*, 6:40–49, 2015.
147. T. Monks, M. Pitt, K. Stein, and M. James. maximizing the population benefit from thrombolysis in acute ischemic stroke: A modeling study of in-hospital delays. *Stroke*, 43(10):2706–2711, 2012.
148. T. Monks, D. Worthington, M. Allen, M. Pitt, K. Stein, and M.A. James. A modelling tool for capacity planning in acute and community stroke services. *BMC Health Services Research*, 16(1):1–8, 2016.
149. A. Najjar, D. Reinharz, C. Girouard, and C. Gagn. A two-step approach for mining patient treatment pathways in administrative healthcare databases. *Artificial Intelligence in Medicine*, 87:34–48, 2018.
150. A. Noro, J.W. Poss, J.P. Hirdes, H. Finne-Soveri, G. Ljunggren, J. Bjrnsson, M. Schroll, and P.V. Jonsson. Method for assigning priority levels in acute care (maple-ac) predicts outcomes of acute hospital care of older persons – a cross-national validation. *BMC Medical Informatics and Decision Making*, 11(1), 2011.
151. B.S.S. Onggo, N.C. Proudlove, S.A. D’Ambrogio, A. Calabrese, S. Bisogno, and N. Levialdi Ghiron. A bpmn extension to support discrete-event simulation for healthcare applications: An explicit representation of queues, attributes and data-driven decision points. *Journal of the Operational Research Society*, 69(5):788–802, 2018.
152. Y.A. Ozcan, E. Tanfani, and A. Testi. A simulation-based modeling framework to deal with clinical pathways. *Proceedings – Winter Simulation Conference*, pages 1190–1201, 2011.
153. Y.A. Ozcan, E. Tnfani, and A. Testi. Improving the performance of surgery-based clinical pathways: a simulation-optimization approach. *Health Care Management Science*, 20(1), 2017.
154. A. Partington, M. Wynn, S. Suriadi, C. Ouyang, and J. Karnon. Process mining for clinical processes: A comparative analysis of four australian hospitals. *ACM Transactions on Management Information Systems*, 5(4), 2015.
155. M.C. Pealoza Ramos, P. Barton, S. Jowett, and A.J. Sutton. A systematic review of research guidelines in decision-analytic modeling. *Value in Health*, 18(4):512–529, 2015.
156. M. Peleg. Computer-interpretable clinical guidelines: A methodological review. *Journal of Biomedical Informatics*, 46(4):744–763, 2013.
157. A. Perer, F. Wang, and J. Hu. Mining and exploring care pathways from electronic medical records with visual analytics. *Journal of Biomedical Informatics*, 56:369–378, 2015.
158. H. Pilgrim, P. Tappenden, J. Chilcott, M. Bending, P. Trueman, A. Shorthouse, and J. Tappenden. The costs and benefits of bowel cancer service developments using discrete event simulation. *Journal of the Operational Research Society*, 60(10):1305–1314, 2009.
159. M. Pitt, T. Monks, S. Crowe, and C. Vasilakis. Systems modelling and simulation in health service design, delivery and decision making. *BMJ Quality and Safety*, 25(1):38–45, 2016.
160. J. Poirier, G.Y. Zou, and J. Koval. Confidence intervals for a difference between lognormal means in cluster randomization trials. *Statistical Methods in Medical Research*, 26(2):598–614, 2017.
161. C. Ponsard, R. De Landtsheer, Y. Guyot, F. Roucoux, and B. Lambeau. Decision making support in the scheduling of chemotherapy coping with quality of care, resources and ethical constraints. *ICEIS 2017 – Proceedings of the 19th International Conference on Enterprise Information Systems*, 1:460–470, 2017.
162. M. Prodel, V. Augusto, X. Xie, B. Jouaneton, and L. Lamarsalle. Discovery of patient pathways from a national hospital database using process mining and integer linear programming. *IEEE International Conference on Automation Science and Engineering*, 2015-October:1409–1414, 2015.
163. S. Rachuba, A. Salmon, Z. Zhelev, and M. Pitt. Redesigning the diagnostic pathway for chest pain patients in emergency departments. *Health Care Management Science*, 21(2):177–191, 2018.
164. M. Ramos-Merino, L.M. lvarez Sabucedo, J.M. Santos-Gago, and J. Sanz-Valero. A bpmn based notation for the representation of workflows in hospital protocols. *Journal of Medical Systems*, 42(10), 2018.
165. W. Rashwan, W. Abo-Hamad, and A. Arisha. A system dynamics view of the acute bed blockage problem in the irish healthcare system. *European Journal of Operational Research*, 247(1):276–293, 2015.
166. O. Rejeb, C. Pilet, S. Hamana, X. Xie, T. Durand, S. Aloui, A. Doly, P. Biron, L. Perrier, and V. Augusto. Performance and cost evaluation of health information systems using micro-costing and discrete-event simulation. *Health Care Management Science*, 21(2):204–223, 2018.
167. F. Rismanchian and Y.H. Lee. Process miningbased method of designing and optimizing the layouts of emergency departments in hospitals. *Health Environments Research and Design Journal*, 10(4):105–120, 2017.
168. E. Rojas, J. Munoz-Gama, M. Seplveda, and D. Capurro. Process mining in healthcare: A literature review. *Journal of Biomedical Informatics*, 61:224–236, 2016.
169. D. Sarno and M.E. Nenni. Daily nurse requirements planning based on simulation of patient flows. *Flexible Services and Manufacturing Journal*, 28(3):526–549, 2016.
170. C.-P. Shen, C. Jigjidsuren, S. Dorjgochoo, C.-H. Chen, W.-H. Chen, C.-K. Hsu, J.-M. Wu, C.-W. Hsueh, M.-S. Lai, C.-T. Tan, E. Altangerel, and F. Lai. A data-mining framework for transnational healthcare system. *Journal of Medical Systems*, 36(4):2565–2575, 2012.
171. N. Shukla, S. Lahiri, and D. Ceglarek. Pathway variation analysis (PVA): Modelling and simulations. *Operations Research for Health Care*, 6:61–77, 2015.
172. N.F. Smedley, B.M. Ellingson, T.F. Cloughesy, and W. Hsu. Longitudinal patterns in clinical and imaging measurements predict residual survival in glioblastoma patients. *Scientific Reports*, 8(1), 2018.
173. B.G. Sobolev, V. Sanchez, and C. Vasilakis. Systematic review of the use of computer simulation modeling of patient flow in surgical care. *Journal of Medical Systems*, 35(1):1–16, 2011.
174. E.M. Soffin and J.T. Yadeau. Enhanced recovery after surgery for primary hip and knee arthroplasty: A review of the evidence. *British Journal of Anaesthesia*, 117:iii62–iii72, 2016.
175. K.W. Soh, C. Walker, and M. OSullivan. A literature review on validated simulations of the surgical services. *Journal of Medical Systems*, 41(4), 2017.
176. L. Standfield, T. Comans, and P. Scuffham. Markov modeling and discrete event simulation in health care: A systematic comparison. *International Journal of Technology Assessment in Health Care*, 30(2):165–172, 2014.
177. A. Stefanini, D. Aloini, R. Dulmin, and V. Mininno. Linking diagnostic-related groups (drgs) to their processes by process mining. *HEALTHINF 2016 – 9th International Conference on Health Informatics, Proceedings; Part of 9th International Joint Conference on Biomedical Engineering Systems and Technologies, BIOSTEC 2016*, pages 438–443, 2016.
178. M. Stuit, H. Wortmann, N. Szirbik, and J. Roodenburg. Multi-view interaction modelling of human collaboration processes: A business process study of head and neck cancer care in a dutch academic hospital. *Journal of Biomedical Informatics*, 44(6):1039–1055, 2011.
179. H. Syed and A.K. Das. Identifying chemotherapy regimens in electronic health record data using interval-encoded sequence alignment. *Lecture Notes in Computer Science (including subseries Lecture Notes in Artificial Intelligence and Lecture Notes in Bioinformatics)*, 9105:143–147, 2015.
180. P. Tappenden, J. Chilcott, A. Brennan, H. Squires, and M. Stevenson. Whole disease modeling to inform resource allocation decisions in cancer: A methodological framework. *Value in Health*, 15(8):1127–1136, 2012.
181. A. Teixeira and M.J.F. De Oliveira. Operations research on hospital admission systems: A first overview of the 2005–2014 decade. *Journal of Physics: Conference Series*, 616(1), 2015.
182. E. Tnfani and A. Testi. A simulation-based decision support tool to analyze clinical pathways in hospital. *International Series in Operations Research and Management Science*, 173:191–211, 2012.
183. A. Tolarczyk and K. Siwek. Sequential pattern recognition for medical records analysis. *Proceedings of 2016 17th International Conference Computational Problems of Electrical Engineering, CPEE 2016*, 2016.
184. S. Tsumoto, H. Iwata, S. Hirano, and Y. Tsumoto. Similarity-based behavior and process mining of medical practices. *Future Generation Computer Systems*, 33:21–31, 2014.
185. K. Uragaki, T. Hosaka, Y. Arahori, M. Kushima, T. Yamazaki, K. Araki, and H. Yokota. Sequential pattern mining on electronic medical records with handling time intervals and the efficacy of medicines. *Proceedings – IEEE Symposium on Computers and Communications*, 2016-August:20–25, 2016.
186. E. Uzun Jacobson, S. Bayer, J. Barlow, M. Dennis, and M.J. MacLeod. The scope for improvement in hyper-acute stroke care in scotland. *Operations Research for Health Care*, 6:50–60, 2015.
187. J. van de Klundert, P. Gorissen, and S. Zeemering. Measuring clinical pathway adherence. *Journal of Biomedical Informatics*, 43(6):861–872, 2010.
188. D.-J. Van Der Zee, M.M.H. Lahr, G.-J. Luijckx, and E. Buskens. Simulation conceptual modeling for optimizing acute stroke care organization. *Proceedings – Winter Simulation Conference*, 2016-February:1403–1414, 2016.
189. R. van Zelm, I. Janssen, K. Vanhaecht, A. de Buck van Overstraeten, M. Panella, W. Sermeus, and E. Coeckelberghs. Development of a model care pathway for adults undergoing colorectal cancer surgery: Evidence-based key interventions and indicators. *Journal of Evaluation in Clinical Practice*, 24(1):232–239, 2018.
190. P.T. Vanberkel, R.J. Boucherie, E.W. Hans, J.L. Hurink, and N. Litvak. Efficiency evaluation for pooling resources in health care. *OR Spectrum*, 34(2):371–390, 2012.
191. S.A. Vanderby, M.W. Carter, T. Noseworthy, and D.A. Marshall. Modelling the complete continuum of care using system dynamics: The case of osteoarthritis in Alberta. *Journal of Simulation*, 9(2):156–169, 2015.
192. V. Vogt, S.M. Scholz, and L. Sundmacher. Applying sequence clustering techniques to explore practice-based ambulatory care pathways in insurance claims data. *European Journal of Public Health*, 28(2):214–219, 2018.
193. J. Walonoski, M. Kramer, J. Nichols, A. Quina, C. Moesel, D. Hall, C. Duffett, K. Dube, T. Gallagher, and S. McLachlan. Synthea: An approach, method, and software mechanism for generating synthetic patients and the synthetic electronic health care record. *Journal of the American Medical Informatics Association*, 25(3):230–238, 2018.
194. F. Walter, A. Webster, S. Scott, and J. Emery. The Andersen model of total patient delay: A systematic review of its application in cancer diagnosis. *Journal of Health Services Research and Policy*, 17(2):110–118, 2012.
195. T. Wang, X. Tian, M. Yu, X. Qi, and L. Yang. Stage division and pattern discovery of complex patient care processes. *Journal of Systems Science and Complexity*, 30(5):1136–1159, 2017.
196. W. Wang, S. Nelson, and J.M. Albert. Estimation of causal mediation effects for a dichotomous outcome in multiple-mediator models using the mediation formula. *Statistics in Medicine*, 32(24):4211–4228, 2013.
197. W. Xu, Y. Zhu, and Y. Geng. Development of an open metadata schema for clinical pathway (opencp) in china. *Methods of Information in Medicine*, 57(4):159–167, 2018.
198. X. Xu, T. Jin, and J. Wang. Summarizing patient daily activities for clinical pathway mining. *2016 IEEE 18th International Conference on e-Health Networking, Applications and Services, Healthcom 2016*, 2016.
199. X. Xu, T. Jin, Z. Wei, C. Lv, and J. Wang. Tcpm: Topic-based clinical pathway mining. *Proceedings – 2016 IEEE 1st International Conference on Connected Health: Applications, Systems and Engineering Technologies, CHASE 2016*, pages 292–301, 2016.
200. X. Xu, T. Jin, Z. Wei, and J. Wang. Incorporating domain knowledge into clinical goal discovering for clinical pathway mining. *2017 IEEE EMBS International Conference on Biomedical and Health Informatics, BHI 2017*, pages 261–264, 2017.
201. X. Xu, T. Jin, Z. Wei, and J. Wang. Incorporating topic assignment constraint and topic correlation limitation into clinical goal discovering for clinical pathway mining. *Journal of Healthcare Engineering*, 2017, 2017.
202. H. Yan, P. Van Gorp, U. Kaymak, X. Lu, L. Ji, C.C. Chiau, H.H.M. Korsten, and H. Duan. Aligning event logs to task-time matrix clinical pathways in bpmn for variance analysis. *IEEE Journal of Biomedical and Health Informatics*, 22(2):311–317, 2018.
203. W.-S. Yang and S.-Y. Hwang. A process-mining framework for the detection of healthcare fraud and abuse. *Expert Systems with Applications*, 31(1):56–58, 2006.
204. X. Yang, R. Han, Y. Guo, J. Bradley, B. Cox, R. Dickinson, and R. Kitney. Modelling and performance analysis of clinical pathways using the stochastic process algebra pepa. *BMC Bioinformatics*, 13(SUPPL 1), 2012.
205. L. Yin, W. Dong, Z. Huang, L. Ji, X. Lv, and H. Duan. On detecting the changes of medical behaviors in clinical pathways. *Chinese Journal of Biomedical Engineering*, 34(3):272–280, 2015.
206. L. Yin, Z. Huang, W. Dong, C. He, and H. Duan. utilizing electronic medical records to discover changing trends of medical behaviors over time. *Methods of Information in Medicine*, 56(MethodsOpen):e49–e66, 2017.
207. S. Yoo, M. Cho, S. Kim, E. Kim, S.M. Park, K. Kim, H. Hwang, and M. Song. Conformance analysis of clinical pathway using electronic health record data. *Healthcare Informatics Research*, 21(3):161–166, 2015.
208. X. Zhang. Application of discrete event simulation in health care: A systematic review. *BMC Health Services Research*, 18(1), 2018.
209. Y. Zhang and R. Padman. Data-driven clinical and cost pathways for chronic care delivery. *American Journal of Managed Care*, 22(12):816–820, 2016.
210. Y. Zhang and R. Padman. An interactive platform to visualize data-driven clinical pathways for the management of multiple chronic conditions. *Studies in Health Technology and Informatics*, 245:672–676, 2017.
211. Y. Zhang, R. Padman, and N. Patel. Paving the cowpath: Learning and visualizing clinical pathways from electronic health record data. *Journal of Biomedical Informatics*, 58:186–197, 2015.
212. M.E. Zonderland, R.J. Boucherie, and A. Al Hanbali. Appointments in care pathways: the *Geo^x^/D/*1 queue with slot reservations. *Queueing Systems*, 79(1):37–51, 2014.
213. A. Zwerling, R.G. White, A. Vassall, T. Cohen, D.W. Dowdy, and R.M.G.J. Houben. modeling of novel diagnostic strategies for active tuberculosis – a systematic review: Current practices and recommendations. *PLoS ONE*, 9(10), 2014.

## Appendix II

**Table A1.** Papers of notable contribution

Reference	Type	Summary	Year Published
[6]	Case Study	Business Process Management technology applied to clinical pathways. Pediatric kidney transplantation case study.	2017
[26]	Case Study	Factors influencing successful adoption of general simulation tools within healthcare organisations.	2013
[28]	I.T.Artifact	Develops a Electronic audit and feedback. (e-A&F) system.	2018
[44]	Evaluation	Telemedicine research.	2006
[106]	Review	Overview of the past, present and future of preoperative cardiovascular care.	2018
[130]	Guidelines and Case Study	Test the feasibility of building full National Institute for Health and Care Excellence (NICE) guidelines models for cost-effectiveness analysis.	2013
[159]	Survey and Case Study	Delivery and design across healthcare planning. A case study in emergency stroke care is presented as an exemplar.	2015
[174]	Narrative Review	Constructing enhanced recovery after surgery (ERAHS) pathways for hip and knee arthroplasty.	2016
[180]	Framework	Developing health economic models of whole systems of disease and treatment pathways for resource allocation decisions.	2012
[193]	I.T Artifact	Synthea - an open-source software package that simulates the lifespan of synthetic patients.	2018
[207]	Conformance analysis	Conformance rates of actual usage of clinical pathway using Electronic Health Record log data.	2015

**Table A2.** Summary of previous literature reviews

Reference	Type	Summary	Dates included	No. Search Engines	No. Papers Included	Major Search Terms
[2]	Literature Review	Model-based cost-utility studies of depression	2002–2010	4	14	Depression, models, computer simulation, cost utility, QALY
[38]	Literature Survey	Data mining for electronic health records (EHRs)	2000–2016	3	2516	Data mining, machine learning, artificial intelligence, mining, AND electronic health record (EHR)
[59]	Literature Review	Mathematical decision models that evaluated 5 α- reductable inhibitors and prostate cancer chemoprevention	until 2013	2	7	Cost-effectiveness, cost-utility, decision-analytic, economic model, AND prostate cancer
[61]	Systematic Review	Clinical pathways	NA	4	22	Clinical pathways, Critical pathways, Care maps, Integrated care pathways, Care pathways
[63]	Systematic Mapping	Process mining in healthcare	2005–2017	10	172	‘Process mining’ in healthcare and clinical pathways
[65]	Systematic Review	Patient-reported outcome measures (PROMs) being utilized by Advanced practice physiotherapists (APPs)	until 2018	5	38	Physio Therapy, Advanced Practice, Patient-reported outcome measures
[69]	Systematic Review	Intraoperative efficacy improvement in surgery	until 2015	1	38	Operating room, surgery, surgical AND Efficiency, Lean, Six Sigma
[77]	Literature Review	Compare adult and pediatric Enhanced Recovery After Surgery (ERAS) pathways	1940–2018	1	NA/83	Enhanced Recovery, Fast Track, surgery AND child
[78]	Literature Review	Process mining in healthcare	until 2016	8	2371	Process mining, healthcare, clinic, hospital, care, health
[84]	Systematic Review	Inpatient bed management	2013–2017	3	92	Bed management, bed assignment, bed planning, bed allocation
[105]	Literature Review	Simulation modelling in stroke care systems	until 2014	1	30	Stroke, simulation, simulation model
[109]	Literature Review	Process mining in oncology	until 2016	5	37	Process mining, data mining, pathway analysis, AND patient flow, AND oncology
[123]	Literature Review	Mathematical modelling for evaluating waiting times in a hospital emergency department	2000–2010	5	29	NA
[132]	Literature Review	Application of generic operational models in health services	until 2013	2	116	Operational model AND health, care, Clinical pathway, Simulation, Markov
[135]	Systematic Review	Data mining and predictive analytics in healthcare operations and supply chain management	until 2015	2	22	Big data, data mining, process mining AND optimizations AND, healthcare
[137]	Literature Review	Multi-appointment scheduling in hospitals	1995–2017	2	56	Multi-appointment, integrated, holistic AND healthcare, AND scheduling
[156]	Literature Review	Computer-interpretable guidelines	2001–2013	1	21	Electronic clinical guidelines, clinical pathway, clinical pathways, care pathway
[155]	Systematic Review	Good practice guidelines and contemporary developments	1990–2014	7	33	NA
[168]	Literature Review	Process mining in healthcare	until 2016	3	74	Process mining, workflow mining, healthcare
[173]	Systematic Review	Simulation of changes in the delivery of surgical care	1957–2007	8	34	Clinical path, patient flow, Markov, system dynamics, discrete event, agent based, statechart
[175]	Literature Review	Validated simulation models on hospital-wide surgical services	2000–2016	4	22	Simulation, AND clinical pathway, care pathway, critical pathway, patient pathway, care map
[176]	Systematic Review	Comparing Markov modelling and discrete event simulation for cost-effectiveness analysis of healthcare technologies	1947–2012	3	22	Discrete event simulation, Markov, microsimulation, Monte-Carlo, economic
[181]	Literature Review	Operations Research applied to Hospital Administration Systems	2005–2014	6	152	Hospital, Admission, Systems
[189]	Literature Review	Evidence based model pathway for surgical patients with colorectal cancer	2006–2014	3	15	Clinical pathway, and colorectal, cancer, enhanced recovery program
[194]	Systematic Review	Application of the Anderson’s Model of Total Patient Delay to asses cancer diagnosis	1979–2009	4	10	NA
[208]	Systematic Review	Discrete event simulation applied to health-related topics and decision making	until 2017	2	211	Discrete event simulation, AND health service, Patient, healthcare
[213]	Systematic Review	Mathematical modelling the cost-effectiveness of diagnostic strategies for active TB	2000–2013	1	36	NA

**Table A3.** Publications categorised by JCR

JCR Category	
MI	[3, 11, 13, 14, 16, 17, 19, 32, 37, 42, 45–47, 52, 54, 56, 57, 66, 72, 73, 1–93, 95–97, 99–101, 108, 122, 124, 131, 140, 144, 149, 150, 157, 160, 164, 170, 178, 187, 196, 197, 206, 211]
OR/MS	[7, 8, 12, 18, 22, 23, 27, 30, 31, 43, 50, 51, 64, 75, 79, 90, 113, 114, 134, 151, 158, 165, 169, 190, 203, 212]
HPS	[9, 10, 21, 33, 39, 40, 80, 102–104, 110, 116, 141, 143, 153, 163, 166, 209]
IE	[34, 120]
AN	[53, 133]
No ISSN	[15, 24, 36, 60, 70, 71, 82, 127, 138, 139, 142, 161, 183, 198–200]

**Table A4.** Way in which information about the pathway was obtained

Obtained	
Data Driven	[5, 7–9, 11, 13, 32, 33, 37, 47–49, 54, 58, 62, 66, 70–72, 74–76, 80, 81, 83, 85–99, 104, 107, 108, 112, 120, 121, 124, 126, 127, 131, 133, 134, 138–140, 143, 144, 147–150, 153, 157, 160, 162, 167, 170, 172, 177–179, 183–185, 191, 192, 195, 197–206, 209–211]
Collaboration	[3, 10, 14, 17, 27, 42, 51, 73, 100, 114, 142, 152, 163]
Both	[12, 20, 35, 40, 56, 101, 103, 129, 136, 146, 154, 166, 171, 186]
Other	[19, 21, 22, 24, 41, 60, 79, 82, 116, 128, 169]

**Table A5.** Clinical condition area of consideration

Condition	Focus	Applied
Acute	[16, 25, 34–36, 46, 50, 60, 66, 110, 111, 114, 136, 145–148, 150, 163, 186, 188]	[5, 15, 29, 37, 42, 64, 70, 71, 79, 88–93, 97, 108, 124, 139, 154, 160, 162, 169, 171, 196, 200–202, 204, 205]
Chronic	[1, 9, 11, 19, 23, 40, 41, 43, 45, 47, 49, 58, 73, 86, 101, 103, 104, 115–117, 122, 158, 161, 172, 179, 191, 192, 209]	[13, 24, 39, 48, 51, 54–56, 81, 82, 85, 94–96, 98, 99, 102, 112, 121, 126, 128, 131, 140, 144, 149, 157, 166, 170, 177, 178, 195, 210, 211]
Surgical	[21, 53, 68, 143, 153]	[20, 32, 57, 62, 107, 125, 133, 152, 185]

**Table A6.** Care level considered

Care Level	
Primary	[4, 11, 16, 19, 23, 25, 27, 40, 43, 110, 111, 114–117, 136, 145, 152, 154, 170, 178, 188, 191, 196]
Secondary	[3, 5, 8, 11–22, 27, 29–36, 41–43, 45, 46, 50–52, 60, 62, 64, 67, 70–76, 79–81, 83, 87–93, 95, 97, 99, 100, 108, 110, 111, 113, 114, 124, 127–129, 133, 134, 136, 138–142, 145–147, 149, 151–154, 158, 160, 162–167, 169–172, 178, 182–188, 190–192, 197–206, 210–212]
Tertiary	[1, 10, 11, 13, 16, 19–21, 32, 47, 53, 57, 68, 73, 82, 85, 94, 96, 98, 99, 102, 103, 107, 112, 121, 125, 126, 133, 140, 145, 148, 152, 154, 158, 161, 166, 177–179, 185, 191, 195, 209]
Disease	[4, 7, 9, 13, 24, 25, 37, 39, 43, 48, 49, 54–56, 58, 66, 86, 101–104, 118–120, 122, 128, 131, 138, 143, 144, 150, 157, 172, 196, 211]
Home Care	[7, 16, 27, 34, 118–120, 142, 145]

**Table A7.** Multiple care levels considered

Multiple Care Levels	
Two	[4, 7, 20, 21, 25, 32, 34, 73, 99, 102, 103, 110, 111, 114, 118–120, 128, 133, 136, 138, 140, 142, 158, 166, 170, 172, 185, 188, 196, 211]
Three	[11, 13, 19, 27, 43, 152, 154, 178, 191]
Four	[16, 145]

**Table A8.** Type of scope considered

Scope	
Hospital	[8, 21, 23, 27, 29–31, 36, 41, 51, 64, 67, 74–76, 79, 85, 108, 111, 114–117, 134, 142, 145–148, 152, 158, 160, 165, 166, 170, 177, 178, 184, 190, 202, 203, 209]
Department	[12, 14, 15, 17, 18, 46, 50, 52, 57, 62, 68, 100, 113, 125, 129, 141, 151, 153, 154, 161, 163, 164, 167, 169, 171, 182, 185, 187, 212]
Clinical	[1, 3, 5, 10, 11, 13, 16, 19, 20, 22, 32–35, 40, 42, 43, 45, 47, 53, 60, 70–73, 80–83, 87–99, 102, 107, 110, 112, 121, 124, 126–128, 133, 136, 139, 140, 149, 162, 179, 183, 186, 188, 191, 192, 195, 197–201, 204–206, 210, 211]
Disease	[4, 7, 9, 13, 24, 25, 37, 39, 43, 48, 49, 54–56, 58, 66, 86, 101–104, 118–120, 122, 128, 131, 138, 143, 144, 150, 157, 172, 196, 211]

**Table A9.** Method

Method	
Data Mining or Machine Learning	[5, 7, 8, 11, 20, 24, 29, 33, 37, 42, 47–49, 54, 62, 66, 70–72, 74, 76, 81–83, 85–94, 96–99, 104, 107, 108, 112, 121, 124, 126–128, 133, 136, 138, 139, 143, 144, 149, 150, 154, 157, 162, 164, 167, 170, 172, 177–179, 183–185, 192, 195, 197–203, 205, 206, 209–211]
Simulation	[1, 3, 7, 9, 10, 12, 15–19, 21, 23, 25, 27, 32, 34–36, 39, 41, 43, 45, 46, 50–53, 60, 64, 67, 68, 73, 79, 100, 102, 108, 110, 111, 113–117, 122, 129, 134, 142, 145–148, 151–153, 158, 160, 163, 165, 166, 169, 171, 182, 186, 188, 190, 191, 196, 204]
Optimisation and Heuristics	[4, 8, 12–14, 18, 22, 30, 31, 54–58, 74–76, 80, 95, 99, 103, 118, 119, 125, 131, 140, 161, 162, 167, 179, 187, 211]
Stochastic Modelling	[7, 11, 40, 101, 120, 140, 141, 151, 190, 203, 211, 212]

**Table A10.** Multiple methods considered

Multiple Methods	
Two	[8, 11, 12, 18, 54, 74, 76, 99, 108, 140, 151, 162, 167, 179, 190, 203]
Three	[7, 211]

**Table A11.** Investigating type

Investigating Type	
Mapping (Ma)	[1, 4, 5, 7, 22, 29, 33, 37, 42, 47, 48, 54, 56, 57, 70, 71, 81, 83, 87, 88, 90, 91, 94–99, 104, 112, 121, 126, 127, 131, 136, 138, 139, 144, 149, 154, 157, 161, 162, 164, 170, 172, 177–179, 183–185, 192, 195, 197–203, 206, 209–211]
Modelling (Mo)	[15, 19–21, 23, 24, 30, 31, 36, 39, 43, 50, 55, 58, 64, 67, 76, 79, 82, 86, 89, 102, 103, 115, 117–119, 122, 129, 145–147, 150, 151, 160, 169, 171, 187, 188, 190, 191, 196, 212]
Improving (I)	[13, 46]
Ma & Mo	[3, 9, 11, 17, 18, 40, 60, 62, 66, 72–74, 80, 85, 90, 93, 101, 107, 108, 114, 120, 124, 125, 128, 133, 134, 140, 143, 158, 205]
Mo & I	[8, 16, 25, 32, 34, 41, 45, 52, 53, 68, 75, 110, 111, 113, 116, 141, 148, 165, 182]
All Types	[10, 12, 14, 27, 35, 49, 51, 100, 142, 152, 153, 163, 166, 167, 186, 204]

**Table A12.** Outcome focus of the pathway

Outcome	
Pathway Mapping	[1, 4, 5, 13, 20, 33, 37, 42, 47, 54–56, 58, 62, 70, 71, 81–83, 86–99, 107, 112, 114, 115, 121, 126–128, 136, 138–140, 149, 154, 157, 162, 164, 171, 172, 177–179, 183, 184, 187, 188, 192, 195, 197–202, 206, 210, 211]
Time	[3, 8, 10, 12, 15, 16, 18, 23, 29–32, 34–36, 41, 46, 50, 52, 57, 62, 64, 67, 68, 72, 74, 75, 80, 85, 94, 100, 108, 110, 118, 129, 133, 138, 141–143, 145–147, 151, 160, 161, 163, 165–167, 169, 182, 185, 190, 204, 205, 212]
Resource	[3, 7, 10, 14, 15, 17, 25, 27, 29, 31, 32, 35, 36, 46, 52, 64, 75, 76, 100, 113, 118–120, 142, 145, 148, 151–153, 160, 161, 163, 170, 182, 191, 204]
Cost	[9, 11, 14, 15, 19, 21, 22, 40, 43, 45, 53, 60, 73, 79, 101, 111, 117, 118, 125, 133, 134, 138, 145, 158, 166, 167, 209]
Patient Progression	[24, 29, 39, 40, 45, 48, 49, 51, 60, 79, 86, 101–104, 107, 116, 122, 124, 131, 139, 144, 145, 150, 186, 196]
Legal	[66, 203]

**Table A13.** Multiple outcomes considered

Multiple Outcomes	
Two	[3, 10, 14, 31, 32, 35, 36, 40, 45, 46, 52, 60, 62, 64, 75, 79, 86, 94, 100, 101, 107, 133, 139, 142, 151, 160, 161, 163, 166, 167, 182, 204]
Three	[15, 29, 118, 138]
Four	[145]

**Table A14.** Planning decision level taxonomy

Decision Level	
Strategic	[3, 8, 9, 14, 16, 19, 22, 25, 27, 29, 31, 32, 39, 41, 43, 45, 46, 49, 52, 60, 64, 67, 72, 73, 80, 101–103, 111, 115, 116, 136, 158, 165–167, 170, 182, 190, 191]
Tactical	[7, 10, 12, 14, 30, 34–36, 68, 74, 76, 79, 86, 108, 113, 117, 141, 142, 145, 147, 148, 161, 163]
Operational	[15, 17, 18, 31, 53, 57, 75, 90, 110, 113, 118–120, 134, 143, 146, 150, 153, 160, 169, 186, 212]
No Decision	[1, 4, 5, 11, 13, 20, 21, 23, 24, 33, 37, 40, 42, 47, 48, 50, 51, 54–56, 58, 62, 66, 70, 71, 81–83, 85, 87–89, 91–100, 104, 107, 112, 114, 121, 122, 124–129, 131, 133, 138–140, 144, 149, 151, 152, 154, 157, 162, 164, 171, 172, 177–179, 183–185, 187, 188, 192, 195–206, 209–211]
